# Progressive 35S promoter methylation increases rapidly during vegetative development in transgenic *Nicotiana attenuata* plants

**DOI:** 10.1186/1471-2229-13-99

**Published:** 2013-07-09

**Authors:** Arne Weinhold, Mario Kallenbach, Ian Thomas Baldwin

**Affiliations:** 1 Department of Molecular Ecology, Max Planck Institute for Chemical Ecology, Hans-Knöll-Straße 8, Jena 07745, Germany

## Abstract

**Background:**

Genetically modified plants are widely used in agriculture and increasingly in ecological research to enable the selective manipulation of plant traits in the field. Despite their broad usage, many aspects of unwanted transgene silencing throughout plant development are still poorly understood. A transgene can be epigenetically silenced by a process called RNA directed DNA methylation (RdDM), which can be seen as a heritable loss of gene expression. The spontaneous nature of transgene silencing has been widely reported, but patterns of acquirement remain still unclear.

**Results:**

Transgenic wild tobacco plants (*Nicotiana attenuata*) expressing heterologous genes coding for antimicrobial peptides displayed an erratic and variable occurrence of transgene silencing. We focused on three independently transformed lines (PNA 1.2, PNA 10.1 and ICE 4.4) as they rapidly lost the expression of the resistance marker and down-regulated transgene expression by more than 200 fold after only one plant generation. Bisulfite sequencing indicated hypermethylation within the 35S and NOS promoters of these lines. To shed light on the progress of methylation establishment, we successively sampled leaf tissues from different stages during plant development and found a rapid increase in 35S promoter methylation during vegetative growth (up to 77% absolute increase within 45 days of growth). The levels of *de novo* methylation were inherited by the offspring without any visible discontinuation. A secondary callus regeneration step could interfere with the establishment of gene silencing and we found successfully restored transgene expression in the offspring of several regenerants.

**Conclusions:**

The unpredictability of the gene silencing process requires a thorough selection and early detection of unstable plant lines. *De novo* methylation of the transgenes was acquired solely during vegetative development and did not require a generational change for its establishment or enhancement. A secondary callus regeneration step provides a convenient way to rescue transgene expression without causing undesirable morphological effects, which is essential for experiments that use transformed plants in the analysis of ecologically important traits.

## Background

Transgenic plants have become an essential component in ecological research, allowing the precise study of gene functions under field conditions [1-3]. Despite progress in the development of more efficient transformation techniques, the unpredictable and stochastic occurrence of transgene silencing and epigenetic alternations after the tissue culture step remain unsolved problems for most plant species [4-7]. Basically two forms of gene silencing have been described, transcriptional gene silencing (TGS), in which gene expression is directly blocked, and posttranscriptional gene silencing (PTGS) in which mRNA is degraded [8]. PTGS has been exploited as a very powerful tool for reverse genetic studies and is revolutionizing plant ecology, particularly for non-model plants, where the introduction of “silencing-constructs” in self-compatible inverted repeat (IR) or antisense (AS) orientations enables the targeted silencing of endogenous genes *in trans*[9-12]. Unfortunately, this process can be undermined by unwanted TGS, if the promoter of the transgene is *de novo* methylated, thereby diminishing the expression of the silencing-construct [13-17]. *De novo* DNA methylation can be highly sequence-specific for a transgene, as a result of the process called RNA-directed DNA methylation (RdDM) [17-20]. However, the pattern of establishment and prerequisites for the methylation process remain elusive [21]. Characteristic symptoms of unwanted transgene silencing are spatially variegated or transient gene expression levels, patterns which have been observed in several different plant taxa including *Nicotiana tabacum*[22-24], *Petunia hybrida*[25,26], *Arabidopsis thaliana*[27,28], *Gentiana triflora* X *G. scabra*[29] and even in some transgenic woody plants such as grapevine (*Vitis* spp.) [30] and birch trees (*Betula platyphylla*) [31].

The wild tobacco (*N. attenuata* Torr. ex S. Watson) is an annual plant, native to the Great Basin Desert in the western United States and is used as a model organism to study traits important for survival under real world conditions, in particular the role of jasmonic acid (JA) in plant defense against herbivores [32]. *N. attenuata* has been frequently transformed with many different sense-expression, inverted repeat (IR) or antisense (AS) silencing-constructs to manipulate different layers of plant defense for field studies of gene function [1,33-37]. A stably transformed plant is only useful for ecological experiments if the transgene-altered phenotype remains stable over the entire period of plant development. In the glasshouse the life cycle of *N. attenuata* takes about 70–80 days until the plant produces seeds and develops from a vegetative rosette-stage, through stalk elongation, into the generative flowering phase. Over the course of development the plant reconfigures its defense strategy from largely inducible to constitutive deployment of various jasmonate-mediated chemical defenses [38]. Transgenerational phenotypic stability is also essential if different lines are to be crossed to combine traits so that parental phenotypes can be faithfully transmitted in a hemizygous state to the subsequent hybrid generations. The *N. attenuata* line ir-ACX1 was created to suppress a particular step in the JA biosynthesis pathway due to the silencing of the endogenous *acetyl-CoA-transferase* 1 (*acx*1), but as recently shown the ability to suppress JA accumulation was lost when T_3_ generation plants were used during a field experiment [37]. Similar findings of leaky or lost phenotypes in *N. attenuata* lines have been reported in other studies [34,36] highlighting the importance of the early detection of “unstable” plant lines.

The methylated form of cytosine was discovered more than 60 years ago [39], but despite the very high amounts found in wheat seedlings, it was long considered only as a “minor base” in plant genomes [40]. Its importance in epigenetic gene regulation is increasingly being recognized, but the overall process remains poorly understood [41-44]. If a genomic sequence functions as a promoter, *de novo* methylation can lead to transcriptional silencing of the downstream gene [45,46]. Cytosine methylation plays an important role in many cellular processes such as tissue-specific gene expression, embryogenesis or genomic imprinting [47]. Nevertheless, its generally accepted main function in plants is in the control of “invasive elements” such as transposons or viral sequences [48-50]. In contrast to mammals, plants not only methylate cytosines in CG dinucleotides, but also in all other possible sequence contexts at CHG and CHH positions (where H = A,T or C) [46,51]. The symmetric DNA methylation patterns at CG and CHG sites can be sustained during semiconservative DNA replication and are therefore somatically and even meiotically stable [52,53]. A methylation at the CHH position is called asymmetric, because it has no mirror position on the complementary DNA strand and hence will be lost during the DNA replication process. For maintenance during mitosis, an asymmetric site needs therefore a constant signal as a permanent *de novo* methylation trigger [18,45,46,54]. Although most aspects of epigenetic inheritance are understood, somatic cells are considered to be relative static and the principles of methylation establishment in vegetative grown plants remain unclear [21,46,55-57].

The aim of this study was to illuminate the timing of the transgene inactivation process and to summarize our strategy for an optimized selection of *N. attenuata* plants with desired, stable phenotypes in a set of antimicrobial peptide expressing lines. Since a combination of TGS and PTGS effects can lead to a progressive shutdown of transgene expression [15,58-60], we were interested in finding early indicators and methods to avoid or even predict unwanted transgene silencing in the wild tobacco *N. attenuata.*

## Results

### Non-Mendelian segregation of the resistance marker as the first indicator of transgene silencing

**Figure 1:**
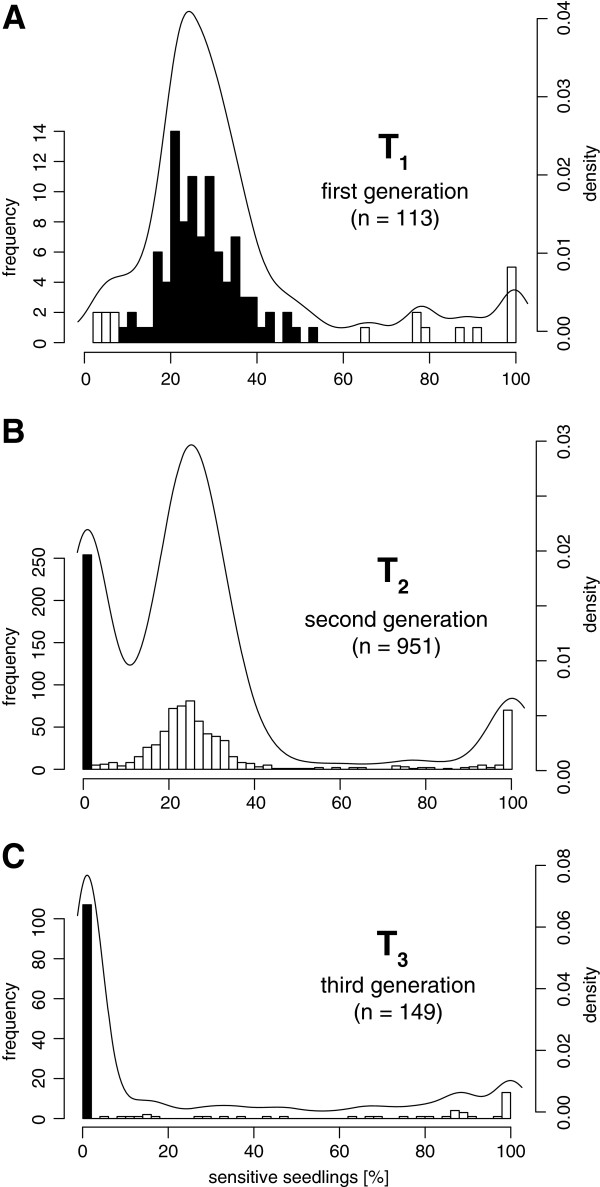
**Segregation analysis of resistance marker loci in *Nicotiana attenuata. *** Histogram of the sensitivity rates of 113 independently transformed *N. attenuata* lines due to segregation of the resistance marker gene monitored over three generations (T_1_–T_3_), overlaid with smoothed density plots. Seedlings with a sensitivity rate between 10–50% were considered to descend from a hemizygous plant. More than 50% sensitivity was interpreted as gene silencing of the hygromycin B resistance marker and a sensitivity rate around 6.25% indicated two independent segregating loci. **A**, Sensitivity rates of T_1_ seedlings collected from 113 independently transformed T_0_ plant lines. Seedlings with the desired sensitivity rate between 10–50% were chosen for further inbreeding (indicated in black). **B**, Sensitivity rates of T_2_ seedlings collected from 951 T_1_ plants. The offspring of nine to ten plants were analyzed per plant line. Descendants from homozygous plants (0% sensitivity) were chosen for further inbreeding (indicated in black). **C**, Sensitivity rates of T_3_ seedlings collected from 149 T_2_ plants homozygous to the transgene. Any occurring sensitivity to the resistance marker was considered as gene silencing. Desired plant lines with sustained resistance were indicated in black.

To be able to work with *N. attenuata* lines that constitutively express antimicrobial peptides under a 35S promoter, we created eleven different transformation vectors containing eleven different antimicrobial peptide genes (see Methods section for details). From each construct more than 10 independent transformed plant lines were created and in total the segregation data of 113 plant lines were observed over three generations of inbreeding. For a high probability in selecting stable expressing plant lines, we used the optimized screening protocol described in Gase *et al.*[61]. This includes the use of flow cytometry (for ploidy analysis), diagnostic PCRs (to confirm completeness of the insert), qRT-PCR (for gene expression analysis) and southern blotting (for detection of insertion number). The segregation analysis of the resistant marker provides not only information about zygosity but can additionally reveal independent segregating loci and the occurrence of unwanted transgene silencing very early in the screening process. A regenerated T_0_ plant should ideally harbor only one T-DNA copy in a single locus, which is usually inherited as a simple, dominant Mendelian trait. According to Mendel’s law of independent assortment [62], offspring derived from self-pollination would show an expected 3:1 ratio, with 25% of the seedlings sensitive to hygromycin. From our segregation data of 113 independently transformed *N. attenuata* lines most of the seedlings showed hygromycin sensitivity in the calculated range (Figure 1). We considered all seedlings with 10–50% sensitivity as being offspring from a hemizygous mother plant and selected only these for further inbreeding (Figure 1A indicated in black). Epigenetic mechanisms could lead to deviations from Mendelian segregation ratios and all seedlings with unusual high numbers of sensitivity (>50%) were therefore excluded from further screening [61]. In the second generation (T_2_), we usually seek seedlings with 0% sensitivity, indicating that they originated from a homozygous plant (Figure 1B). As a selection criterion, all sibling plants of the same line should not deviate from any of the expected ratios and show also 0% or 10–50% sensitivity. The occurrence of a single plant with non-Mendelian segregation (>50%) would lead to an exclusion of the entire transgenic line. In the T_3_ generation, the progenies from the homozygous plants were again tested for stability and any newly occurring segregation led to the exclusion of the line (Figure 1C).

The majority of seedlings of the more than 1200 analyzed plants segregated within the expected ranges, nevertheless 12 of 113 lines (11%) were excluded in the T_1_ stage, 22 of 94 (23%) in the T_2_ stage and 15 of 70 (21%) in the T_3_ stage, due to non-Mendelian segregation patterns. Altogether 43% of the antimicrobial peptide expressing *N. attenuata* lines were excluded for this reason. The T_3_ seedlings from three independent lines (PNA 1.2, PNA 10.1 and ICE 4.4.) indicated nearly a complete loss of resistance, with sensitivity rates comparable to wild-type seedlings. Because of this drastic and uniform switch within only one plant generation, these three lines provided the opportunity to further investigate the otherwise unpredictable occurrence of gene silencing.

### Variability in transgene expression precedes loss of resistance

**Figure 2:**
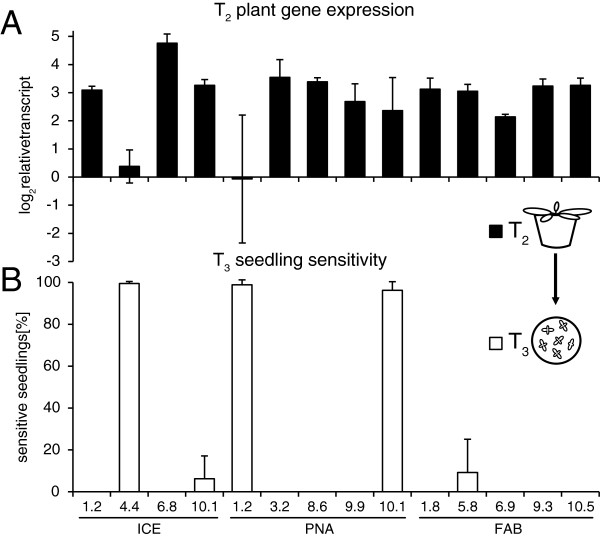
**Comparison of transgene expression and the subsequent loss of hygromycin resistance. A**, Gene expression analysis in homozygous plants of 14 independent *N. attenuata* lines expressing three different antimicrobial peptides (PNA, ICE and FAB). Transcript abundance was determined by qRT-PCR on cDNA of rosette-stage leaves in T_2_ plants. Bars indicate the Δ*C*_T_ mean expression (log_2_ fold expression) relative to actin as the reference gene (±SD, n = 3 plants). The independent transgenic lines showed different strengths of transgene expression, with occasionally low or variable pattern in certain lines. **B**, Hygromycin sensitivity of T_3_ seedlings (direct descendants of the plants used for gene expression). Per plant, 60 seeds were germinated on a hygromycin containing GB5 medium (±SD, n = 3 plants). Homozygous plants should be fully resistant and show 0% sensitivity. The X-axis indicates line number and genotype.

To select appropriate transgenic lines with high levels of transgene expression, we routinely analyze homozygous T_2_ plants by qRT-PCR during the screening process. As an example, we show the transgene expression profiles for three antimicrobial peptides (PNA, ICE and FAB) in 14 independently transformed *N. attenuata* lines (see Methods section for details). Several plant lines showed the desired high and uniform levels of gene expression, whereas others showed low or variable gene expression levels among independently transformed lines expressing the same constructs (Figure 2A). The offspring of these plants was tested on hygromycin containing media, to confirm enduring resistance within the T_3_ seedlings (Figure 2B). Of particular interest were lines ICE 4.4, PNA 1.2 and PNA 10.1, because they nearly completely lost hygromycin resistance in the T_3_ generation and before this, showed even variable expression of the antimicrobial peptide genes in the T_2_ (Figure 2).

**Figure 3:**
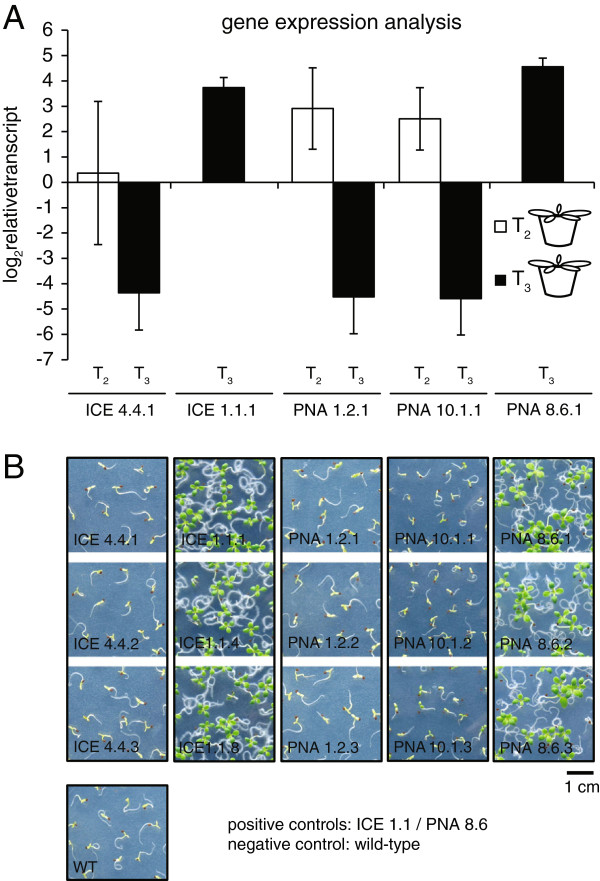
**Progressive loss of transgene expression in subsequent generations. A**, Gene expression analysis in homozygous plants of 5 independent *N. attenuata* lines, comparing two consecutive generations (T_2_ and T_3_). Transcript abundance of the ICE and PNA transgenes was determined by qRT-PCR on cDNA of rosette-stage leaves (30 dpg). Bars indicate the Δ*C*_T_ mean expression (log_2_ fold expression) relative to actin as the reference gene (±SD, n = 3 plants). The T_3_ generation of the lines ICE 4.4.1, PNA 1.2.1 and PNA 10.1.1 indicated a strong reduction of transgene expression. Lines ICE 1.1.1 and PNA 8.6.1 showed stable and high transgene expression in the T_3_ generation. **B**, Photographs of 15-day-old T_3_ seedlings grown on a hygromycin B containing GB5 medium. Sensitivity to hygromycin was indicated by small, yellowish seedlings and the lack of root hairs. Resistant seedlings were larger, with dark green cotyledons and clearly developed root hairs. The lines ICE 4.4.1, PNA 1.2.1 and PNA 10.1.1 showed sensitivity to hygromycin in the T_3_ generation similar to wild-type, whereas the lines ICE 1.1.1 and PNA 8.6.1 remained hygromycin resistance. For each line seeds of three different plants were analyzed and wild-type seeds used as negative control.

To analyze how much a complete loss of resistance correlates with the downregulation of the neighboring transgene, we compared expression levels in both generations (T_2_ and T_3_) from lines PNA 1.2, PNA 10.1 and ICE 4.4. To ensure similar growth conditions, the germination was performed on hygromycin-free media and lines showing stable transgene expression (PNA 8.6.1 and ICE 1.1.1) were included as positive controls. The expression analysis again revealed a very high plant-to-plant variability in the T_2_ stage (Figure 3A), but also a very strong down-regulation of gene expression in the T_3_ generation. This was consistent with the observed loss of hygromycin resistance (Figure 3B). Comparing the T_2_ and T_3_ stage, plants of line ICE 4.4.1 showed a 41 fold (10–73), lines PNA 1.2.1 a 268 fold (63–472) and lines PNA 10.1.1 a 210 fold (51–370) reduced transgene expression, respectively (Figure 3A, Additional file 1). Compared to the stable expressing control lines (PNA 8.6.1 and ICE 1.1.1), the results of the transgene silencing were much more apparent and line ICE 4.4.1 showed 428 fold (99–757), line PNA 1.2.1 836 fold (197–1476) and PNA 10.1.1 872 fold (210–1534) lower gene expression levels, respectively (Additional file 1). In summary, transgenic lines that indicated a loss of hygromycin resistance had an at least 100 fold lower transgene expression, compared to a stable lines expressing the same constructs.

### Multiple T-DNA insertions in silenced lines

**Figure 4:**
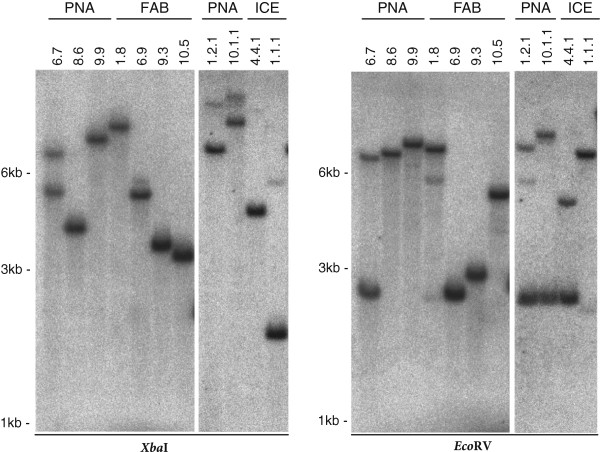
**Southern blot analysis of transgenic *N. attenuata *plants.** Southern blot hybridization analysis was performed to detect the T-DNA copy number in the transgenic plant lines. Genomic DNA was isolated from homozygous seedlings and digested overnight in separate reactions with *Xba*I or *Eco*RV. A radiolabeled fragment of the hygromycin resistance gene (*hptII*) served as probe. The blot indicates the presence of two T-DNA insertions for lines PNA 6.7, PNA 1.2.1, PNA 10.1.1, ICE 4.4.1 and ICE 1.2. The fragment size from the DNA marker is indicated.

Several reports describe a correlation between the incidence of unwanted gene silencing and high transgene copy number, making the selection of single copy T-DNA insertions by Southern blotting a very important part of the screening process [61]. The Southern blot analysis of the silencing affected lines ICE 4.4.1, PNA 1.2.1 and PNA 10.1.1 indicated in the *Xba*I digest no evidence for abnormalities, but the digest with *Eco*RV indicated two T-DNA insertions for all three lines (Figure 4). Unusually, the second T-DNA fragment showed nearly the same size in all three independently transformed lines. Since the fragment size resembles the size of the entire transgenic cassette from left to right border (2.84 kb for pSOL9PNA and 2.76 kb for pSOL9ICE respectively) this indicates the integration of two T-DNA copies adjacent to each other, which could be responsible for the observed transgene silencing in these lines. However, multiple T-DNA copies at two independent loci can be also identified much earlier in the screening process by their unusual segregation rate (6.25% sensitive seedlings instead of 25% for a single locus). In our dataset, only a very small portion of lines (6 out of 113) showed a segregation rate around 6.25% in the T_1_ stage and were considered as harboring transgenes at two independent loci (Figure 1A). This enables an early exclusion of these lines from the further screening process. The Southern blot indicated for most of the analyzed lines only single T-DNA insertions, including the stable control lines (ICE 1.1.1 and PNA 8.6) (Figure 4).

### Sensitive seedlings showed increased NOS promoter methylation

**Figure 5:**
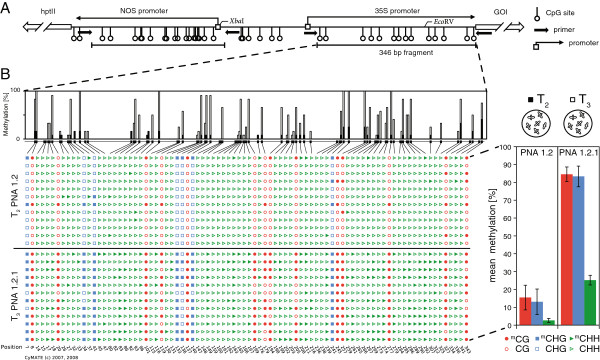
**Schematic illustration of the promoter region and the methylation frequencies within the 35S promoter. A**, Within the transfer DNA (T-DNA) the resistance marker (*hpt*II) is driven by a *nopaline synthase* (NOS) promoter and confers resistance to the antibiotic hygromycin B. The gene of interest (GOI) is driven by a cauliflower mosaic virus promoter (35S). For methylation analysis a 294 bp fragment of the NOS promoter or a 346 bp fragment of the 35S promoter were amplified with the indicated bisulfite primers. Positions of the restriction sites of *Xba*I and *Eco*RV are indicated. **B**, Graphical output of the 35S promoter wide methylation in two consecutive generations of line PNA 1.2 (T_2_ and T_3_ stage). DNA was isolated from seedlings 15 days post germination (dpg). Filled symbols indicate cytosine methylation, whereas empty symbols indicate a lack of methylation. Red circles represent CG sites, blue squares represent CHG sites and green triangles represent CHH sites, whereas H = A,T or C. Analysis was performed with CyMATE [127]. The upper part illustrates the spatial distribution of the methylation sites within the promoter sequence. Mean cytosine methylation was calculated for the three methylation sites (CG, CHG and CHH) from individually picked clones (±SEM, n = 12 clones).

Unwanted or unintended transgene silencing was commonly associated with an increase in methylation within the promoter region of the transgene [24,26,29,63]. Since we found evidence for epigenetic gene silencing (intermediate stages of sensitivity and high variability among replicates), we analyzed promoter methylation levels in the transgenic cassette by bisulfite sequencing. Seedlings from line ICE 10.1 showed a transitional loss of hygromycin resistance and we separated hygromycin sensitive (yellow) and hygromycin resistant (green) seedlings to compare NOS promoter methylation levels within a 294 bp fragment (Figure 5A, Additional file 2). Among these isogenic seedlings, the resistant phenotypes were consistent with the methylation levels and sensitive seedlings had increased methylation levels, particular in the CHG and CHH sites (Additional file 2). Interestingly, the CTG at the 84th position (123 bp before translation start site) was entirely methylation free in resistant seedlings, but to 100% methylated in sensitive seedlings (Additional file 2A). Since this site is located directly downstream of a CCAAT box [64] it appears to be particularly important for the transcription process.

### Hypermethylation of the 35S promoter

For the methylation analysis of the 35S promoter, individual reverse primers were designed for the two different expression cassettes (pSOL9PNA, pSOL9ICE) which allowed amplification of nearly the entire 35S promoter sequence (Figure 5A). Within the 346 bp fragment a total of 14 CG, 7 CHG and 65 CHH sites were found as potential targets for methylation. To allow the direct comparison of promoter methylation differences in (resistant) T_2_ and (sensitive) T_3_ seedlings, all seeds were germinated on hygromycin-free media. The analysis of line PNA 1.2 indicated in the T_2_ stage seedlings methylation levels of 15.5% (±6.9%) CG and 13.1% (±7.1%) CHG methylation, respectively (Figure 5B). In comparison, the methylation rates of T_3_ stage seedlings (PNA 1.2.1) were more than 5 fold increased with 84.5% (±4.1%) CG and 83.3% (±5.8%) CHG methylation. At the asymmetric sites, the CHH methylations levels were 9 fold increased from 2.7% (±1.2%) in T_2_ to 25.3% (±2.7%) in T_3_ seedlings. The clearly increased levels of 35S promoter methylation were consistent with the observed loss of gene expression in this generation (Figure 3A).

### *De novo* cytosine methylation is only acquired during vegetative growth

**Figure 6:**
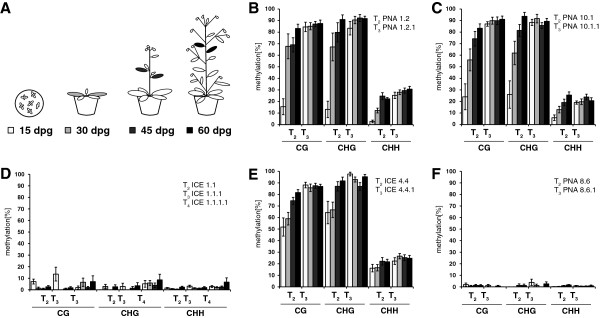
**Changes in 35S promoter methylation during vegetative development. A**, Developmental stages of *N. attenuata* plants used for DNA isolation. The time of harvest is described as days post germination (dpg). Tissues were harvested from cotyledons and first true leaves of seedlings (15 dpg), leaves from rosette-stage plants (30 dpg), cauline leaves from elongating plants (45 dpg) and cauline leaves from flowering plants (60 dpg). **B-F**, Mean cytosine methylation of the 35S promoter among plant development, separately shown for the individual sites (CG, CHG and CHH). The lines ICE 4.4, PNA 1.2 and PNA 10.1 showed increasing methylation levels in the 35S promoter, whereas the stable expressing control lines (ICE 1.1 and PNA 8.6) showed low methylation levels (±SEM, n = 10-12 clones). For the detailed data from individual clones see Additional file 3.

**Figure 7:**
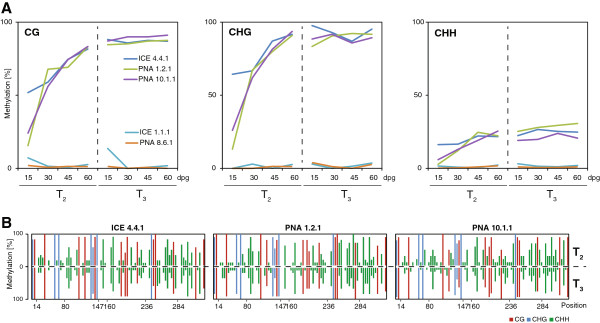
**Progression of the 35S promoter methylation over two generations. A**, Mean cytosine methylation values from Figure 6 shown as trend lines over time. The methylation increase across plant development follows a similar trend in the three independent lines (ICE 4.4, PNA 1.2 and PNA 10.1). The control lines (ICE 1.1 and PNA 8.6) showed no increase in cytosine methylation over time. The generational change is denoted by the dashed line (dpg = days post germination). **B**, Methylation frequencies of individual positions across the generational change. Sequence wide comparison of the T_2_ stage (60 dpg) with the T_3_ stage (15 dpg) in the independent lines (ICE 4.4, PNA 1.2 and PNA 10.1). Methylation rates at individual positions were sustained across the generational transition. Positions with particular low CHH methylation frequency were indicated.

To trace methylation changes of the 35S promoter at different times during plant growth, we sequentially sampled leaf material 30, 45 and 60 days post germination (dpg) (Figure 6A). The three silencing affected lines (PNA 1.2.1, PNA 10.1.1 and ICE 4.4.1) showed in both generations (T_2_ and T_3_) much higher 35S promoter methylation rates compared to the control lines (ICE 1.1.1 and PNA 8.6.1) (Figure 6). The line PNA 8.6.1 indicated the lowest methylation levels and showed throughout the sampling period over both generations mean rates of only 0.9% (±0.7%) in CG, 1.3% (±1.0%) in CHG and 0.8% (±0.3%) in CHH methylation (Figure 6F, Additional file 3). These extremely low methylation levels indicate a complete DNA conversion during the bisulfite treatment and therefore a negligible false positive signal due to incomplete conversion. The second control was investigated until the T_4_ generation (ICE 1.1.1.1) and showed in all three generations consistent low rates of 35S promoter methylation (Figure 6D). These two stable expressing lines indicated no tendency for an increase in 35S promoter methylation after a generational change or during vegetative growth (Figure 6, Figure 7A).

In contrast, the unstable lines ICE 4.4, PNA 1.2 and PNA 10.1 all showed increasing levels of 35S promoter methylation during growth (Figure 6B,C,E; Figure 7A). As a consequence the methylation levels deviated strongly between seedlings and flowering plants within the same generation. For instance, the CHG methylation levels of line PNA 1.2 indicated only 13.1% (±7.1) in seedlings but 90.9% (±4.0%) in flowering plants. This resembles an absolute methylation increase during plant development of more than 77% within only 45 days. The most rapid cytosine methylation increase was observed between seedlings and rosette-stage plants, where the CG and CHG levels changed within 15 days with a velocity of more than 3% per day (Figure 7A). Although the ICE 4.4 line started initially with higher methylation levels in seedling stage, it followed a similar trend and all three independent lines showed a similarly dramatic increase in methylation over time (Figure 7A). During the growth of T_3_ plants, the promoter methylation levels increased only slightly and reached a plateau-like level at around 90% for CG and CHG sites and ca. 30% for CHH sites.

Surprisingly, at the generational transition low differences could be observed between the T_2_ and T_3_ plants. The mean methylation levels of the T_3_ seedlings were highly similar to the levels found in the flowering T_2_ plants (Figure 7). Even the comparison of the individual frequencies at the individual positions indicated no methylation resetting or enhancement across the generational change (Figure 7B). It should be explicitly mentioned that the T_3_ generation seeds were not collected from exactly the same plants used here as T_2_ generation. Both seed generations had been collected beforehand and both generations were grown simultaneously adjacent to each other in the glasshouse. Regardless, the intensity of the methylation increase was highly reproducible and the patterns from both generations matched perfectly (Figure 7A). Among all analyzed clones certain asymmetric positions were only methylated at very low frequencies. In particular, the cytosines at the 14th and 160th position showed, for instance, 0% methylation in both generations (Figure 7B). We grouped the asymmetric CHH sites into low, medium and high methylated positions and found that the overall methylation preference was nucleotide-specific with higher probability at certain positions (e. g. CAA) compared to others (e. g. CCC) (Additional file 4). These findings were similar to the site-specific preferences of asymmetric positions found in a genome-wide analysis of the epigenome in Arabidopsis [65].

### The epigenetic status of the transgene was equally inherited by parental lines

Since we commonly combine phenotypes of transgenic plants by crossing, we wanted to determine whether the heredity of a silenced transgene might be parent-of-origin-specific. We performed reciprocal crosses between wild-type and transgenic lines and tested the hemizygous offspring for hygromycin resistance. The crosses with the silencing affected lines (PNA 1.2, PNA 10.1 and ICE 4.4) all showed high levels of hygromycin sensitivity, independent of the direction of the cross revealing equal inheritance of the silenced allele through both female and male gametes (Additional file 5). The crossings with the stable expressing control lines (PNA 8.6.1 and ICE 1.1.1) always retained their hygromycin resistance. Although crossings had, in certain cases, the potential to reduce silencing [28,66], we did not observe a reduction compared to plants produced from self-pollinations.

### Equivalent transgene inactivation in IR-lines

Unwanted transgene inactivation is not restricted to sense expression lines and has been reported frequently for inverted repeat (IR) constructs, which can also lose their *in trans* silencing ability [15,67]. In the process of producing several hundred IR-lines for the targeted silencing of endogenous *N. attenuata* genes involved in plant defense against herbivores, we have observed several incidents of resistance marker loss in several IR-lines over the past decade. Most recently, this was observed in the T_3_ generation of the ir-ACX1 line, which normally shows a reduced ability to accumulate jasmonic acid (JA) after wounding due to the *in trans* silencing of the endogenous *acx*1 gene [37]. Consistently with our previous observations, the T_3_ seedlings of ir-ACX1 also lost the ability to grow on hygromycin containing media (Additional file 6AB). To test the general applicability of a cell-culture induced transgene reactivation we included this IR-line as a candidate for the secondary regeneration process.

### Restored gene expression after secondary regeneration

**Figure 8:**
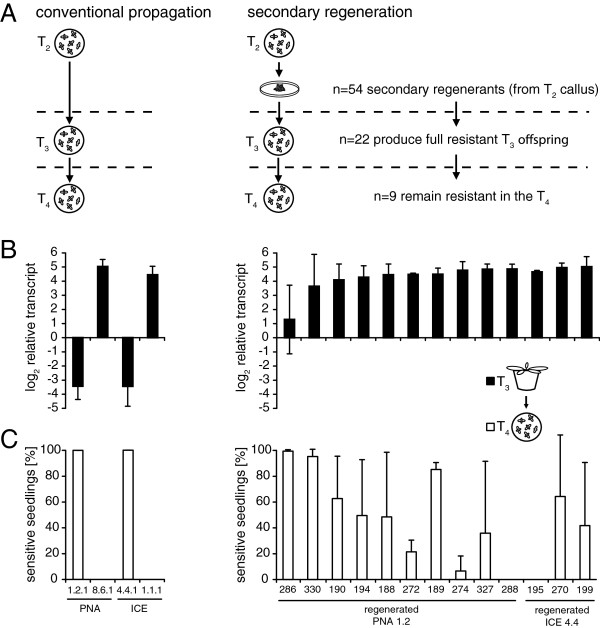
**Recovery of transgene expression after secondary regeneration. A**, Secondary callus regeneration was performed from hypocotyls of homozygous T_2_ seedlings from the lines PNA 1.2, ICE 4.4 and ir-ACX1. From the 54 secondary regenerated plants 22 produced offspring which showed full resistance to hygromycin. In the T_4_ stage the offspring from 9 lines remained resistant. **B**, Gene expression analysis after secondary regeneration. Fully resistant offspring were grown in the glasshouse and qRT-PCR was performed on leaf cDNA from rosette-stage plants. For each genotype conventionally propagated T_3_ plants were included as positive and negative controls, respectively. Bars indicate the Δ*C*_T_ mean expression (log_2_ fold expression) of the ectopically expressed PNA and ICE transgenes relative to actin as the reference gene (±SD, n = 3 plants). **C**, Hygromycin sensitivity of T_4_ seedlings (direct descendants of the plants used for gene expression) indicates an ongoing silencing process (±SD, n = 3 plants).

Most methods used to arrest the progress of transgene silencing include the use of cytidine analogs or viral methylation inhibitors, but these substances can cause severe growth and developmental abnormalities [68]. The cell culture step of the plant transformation procedure itself has been shown to be a significant source of methylation changes [4,69,70] and we evaluated whether the addition of a secondary cell culture step could interfere with the somatic acquirement of *de novo* methylation for the recovery of phenotypes in transformed but epigenetically silenced *N. attenuata* lines. Explant cultures were created from hypocotyls of transgenic homozygous T_2_ seedlings of lines PNA 1.2, ICE 4.4 and ir-ACX1 and were called “secondary regenerants”. The offspring of the secondary regenerants showed a large variability in hygromycin resistance. Most strikingly 41% of the regenerated plants produced offspring with full resistance to hygromycin and only 24% showed a resistance loss as seen after conventional propagation of these lines (Figure 8A, Additional file 7A).

To test if these phenotypically “recovered” plants also were restored in the expression of the transgene, we isolated RNA from rosette-stage plants. The gene expression analysis indicated much higher gene expression levels (about 200–300 fold increased) after the secondary regeneration, compared to conventionally propagated plants of the same line (Figure 8B). Most of the regenerated lines now showed gene expression levels very similar to those of the stable expressing lines (PNA 8.6.1 and ICE 1.1.1). The transgene activity of the ir-ACX1 line was not tested by gene expression analysis, but instead we determined the ability to suppress JA accumulation after simulated herbivory, as it would be performed during an experiment. All offspring from the tested ir-ACX1 regenerants showed suppressed JA accumulation, compared to wild-type plants (Additional file 6C). This indicated an actively expressed IR-construct and a recovered *in trans* silencing ability of the endogenous *acx1* gene after secondary regeneration.

To explore the durability of this recovery, we germinated the subsequent generation (T_4_) on hygromycin containing media. Here the progression of marker gene silencing returned with the characteristic highly variable plant-to-plant pattern of hygromycin sensitivity (Figure 8C, Additional file 6D, Additional file 7B). Lines with low or variable gene expression levels had the highest probability of losing the resistance in the subsequent generation indicating a negative correlation between strength of transgene expression and the subsequent loss of the resistance marker. Finally, at least one line from each of the PNA and ICE regenerants (No. 288 and 195), but seven of the ir-ACX1 regenerants (No. 170, 174, 176, 185, 263, 264 and 265) showed enduring resistance up to the T_4_ generation.

## Discussion

### Erratic occurrence of unwanted transgene silencing

This study summarizes our experience in the overall occurrence of transgene silencing during the screening of *N. attenuata* plants and provides guidance in identifying and avoiding unstable plant lines. Erratic occurrence and variegated phenotypes are commonly reported phenomena of transgene silencing and have been shown in many different plant species [22,25,27,29,71]. This was recently illustrated for *N. benthamiana* plants, transformed with a 35S:GFP construct [58,59]. These plants showed erratic and non-uniform *gfp* expression phenotypes, which differed strongly among isogenic sibling plants, but also among tissues from the same plant. If no visual marker is used, as in our case, the accurate selection based on the resistance marker turns out to be extremely important. Here, the miscellaneous inactivation pattern could be found in the intermediate resistance stages of seedlings or so called “gradual silencing” [27,71,72]. We frequently found intermediate resistant seedlings together with a non-Mendelian distribution (which could also strongly differ among sibling plants). We hypothesize that the gene silencing starts in the 35S promoter and then gradually spreads into the NOS promoter of the resistance marker, as discussed in Mishiba *et al.*[63]. Here the advantage of a head-to-head orientation of both promoters becomes clear, as it places them in close vicinity and a loss of the resistance marker would provide an accurate harbinger of the forthcoming silencing within the expression cassette.

**Figure 9:**
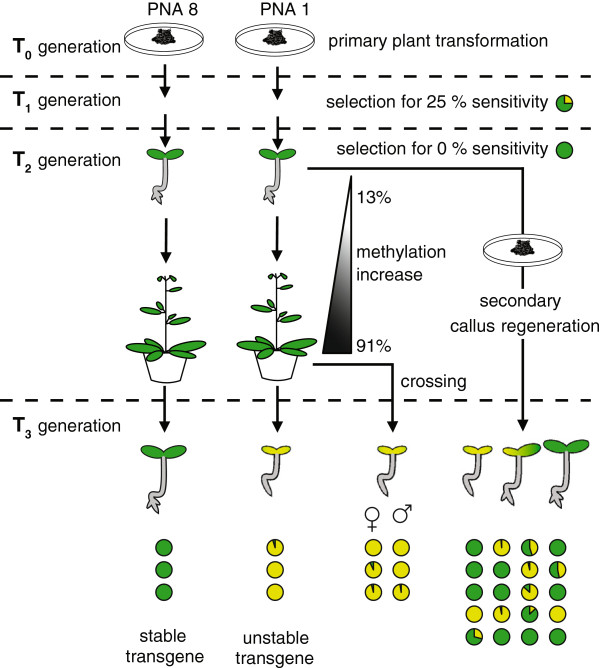
**Overview and summary of the findings.** Transgenic *N. attenuata* lines can entirely loose hygromycin resistance and transgene expression within a single generation. Increase of cytosine methylation levels within the 35S promoter were acquired only during vegetative growth, resulting in higher methylation levels in later developmental stages. The silencing of the resistance marker was equally distributed to subsequent generations after reciprocal crossings with wild-type plants demonstrating a lack of parental bias in the transition of the transgene silencing. The acquisition of transgene silencing could be bypassed with a secondary callus regeneration step, resulting in variable levels of resistance and a recovered transgene expression in the offspring. The pie charts represent the percentage of sensitive and resistant seedlings (data derived from line PNA 1.2.1).

The following three indicators were our major criteria for the early detection of plant lines affected by unwanted gene silencing: (A) unusual segregation rates with >50% of sensitive seedlings, (B) intermediate phenotypes of seedlings with unclear levels of resistance and (C) large differences in gene expression among isogenic plants. We suggest from our experience that testing the subsequent generations for resistance would be the easiest way to ensure stable transgene expression in *N. attenuata*. It is generally advisable to keep the number of generations as small as possible in transgenic plants, since with each new generation the probability of silencing increases. These selection criteria are independent of the mechanism responsible for the transgene silencing process, whether it be by TGS or PTGS [73-75]. As long as the selected plant lines show uniform levels of gene expression and Mendelian pattern of inheritance for the resistance marker, they could be considered as “stable” and used for further experiments (Figure 9).

### Sense transgene silencing in *Nicotiana attenuata*

The intensity of transgene silencing can vary greatly among different plant species. In transgenic gentian plants (*Gentiana triflora* X *G. scabra*) the 35S enhancer sequence showed progressive methylation, independently of copy number and position of insertion [63]. All tested gentian lines showed strong *de novo* methylation, whereas the same construct was methylated with much lower rates in *N. tabacum*[63]. Even among closely related *Nicotiana* species the spontaneous silencing of a transgene was associated with higher methylation levels in *N. benthamiana* than in *N. tabacum*[58]. Similar observations were made with unstable transgene expression in *N. plumbaginifolia*[76]. These reports are consistent with the hypothesis of a more rigorous gene silencing machinery in wild diploid plant species, than in the cultivated tetraploid crop. Gene silencing by DNA methylation is a natural defense mechanism against viruses, transposons and other form of “invasive elements” [47,48,50]. Plants have a more complex and sophisticated gene silencing apparatus than animals do and make use of cytosine methylation at multiple sites in combination with histone modifications and harbor a vast variety of small RNAs [44,46]. Plants even have a signal transmission pathway for small RNAs, which can act as mobile signals to direct RdDM systemically [77]. The very active systemic spreading of the silencing signal through the phloem was first observed in the solanaceous plants, tobacco and tomato [78,79] and later demonstrated also for Arabidopsis [80]. From our initial 113 independent sense expression *N. attenuata* lines we omitted 43% after three generations due to indications of gene silencing. *N. attenuata* has a highly sophisticated suite of defenses against herbivores [32] and it might be, that this plant also has an active methylation apparatus to protect its genome against genetic manipulations [81], which Michael Wassenegger once aptly called a “gene silencing-based resistance against transgene overexpression” [82].

### Factors influencing transgene silencing – the gene dosage effect

Factors which have often been shown to increase the probability of transgene silencing are the transgene copy number and the strength of expression [83,84]. In addition, T-DNA rearrangements, read-through transcripts or improperly terminated or non-polyadenylated mRNA are also associated with transgene silencing [85-87]. Certainly position effects and integration into heterochromatin have been frequently reported in association with local gene silencing [26,88], but integrations into euchromatin can be similarly silenced and more recent studies suggest that overall, the insertion position plays only a minor role [89,90]. Strong viral promoters, such as the 35S cauliflower mosaic virus promoter, were thought to produce “aberrant RNA” after exceeding a certain threshold of expression [16,85]. The progressive methylation of the 35S promoter and the observed downregulation of the transgene in the lines ICE 4.4, PNA 1.2 and PNA 10.1 might be mainly induced by the presence of two T-DNA copies in close proximity to each other (Figure 4). These complex insertions at a single locus can trigger transgene silencing as shown in earlier studies [91]. Any form of repeated T-DNA arrangement appears to increase the overall silencing probability [28,92]. But despite the intensity, the methylation increase occurred relatively late in these lines and the loss of the resistance marker was not revealed until the T_3_ generation (Figure 2B). We hypothesize that the expression of the two T-DNA copies remains below a threshold level when plants are hemizygous. Once homozygous in the T_2_, these thresholds are exceeded and the sum of the four T-DNA copies likely initiate the silencing process. This scenario would explain why also all hemizygous T_2_ sibling plants of these lines were inconspicuous and showed no abnormal segregation.

The attempt to reactivate a silenced transgene by crossing with wild-type plants, to create hemizygous offspring with reduced T-DNA copy number, was partly successful in Arabidopsis and petunia [28,66]. In our case the sensitivity in crossings did not differ from self-pollination, probably because the methylation levels had already accumulated past the silencing threshold in flowering T_2_ plants (Figure 9). Similar as reported for *N. tabacum* hybrids, we found no evidence of a specifically maternal or paternal contribution to the inactivation process [71]. Further monitoring of the crosses could be still interesting if after ongoing propagation, demethylation might occur, as has been seen in other backcrosses with wild-type plants [24].

### Successive increase of *de novo* methylation during development

Usually, epigenetic modifications were considered to be stable in somatic cells and during normal plant development [46,56]. Most substantial epigenetic changes have been reported during gamete formation and embryogenesis in plants [93-95]. Progressive demethylation events that could be observed in endosperm tissue were interpreted as a way to reinforce transposon methylation in the embryo [96-98]. Since transgene silencing has been often described as a sudden switch of the phenotype between plant generations, a similar mechanism might have been responsible for enhancing transgene methylation during the reproductive phase. Our observation of a high variability in rosette-stage plants (line PNA 1.2 showed 23 fold difference in gene expression among biological replicates; Figure 2A) lead to the hypothesis that epigenetic changes might start already early during vegetative growth and increase with different velocities amongst individual plants. Other studies suggested a somatic inactivation as well, pointing to evidence of diminishing expression of a reporter gene during development [71,99,100]. However, in these studies, methylation levels were not analyzed in different stages of plant development. Our methylation kinetic showed a strong somatic increase during growth, but nearly no changes between the generations, resembling a continuous inheritance of the methylation status to the offspring (Figure 7). The recent model of a methylation reinforcement during the reproductive stage, as seen for transposons [98], seems to be not applicable to the *de novo* methylation of transgenes.

Successive analysis of methylation changes have largely been restricted to tissue cultures or micropropagated plants [101]. In a long-term callus cultures of pearl millet (*Pennisetum glaucum*), a gradual decrease in GUS activity could be associated with increased methylation levels, 18 month after transformation [102]. In potato, a successive increase of gene silencing could be shown during a 5 year period of vegetative propagation [84]. In contrast, we found within only 15 days of normal plant development an absolute increase of 50% in total CG methylation. Developmental methylation increases reported in flax and Arabidopsis were only observed after treatment with DNA demethylating agents and therefore more a remethylation to the former status [103,104]. Likewise, the demethylated genome of Arabidopsis *ddm1* mutants showed remethylation after complementation with the wild-type allele [105]. However, it required multiple generations to reach approximately wild-type levels and methylation changes in different plant stages were not compared. A plant stage dependent transgene expression is particular problematic if certain phenotypes (e.g. flower movement) can be only observed in late developmental stages. For ecological field experiments in which plant fitness measurements play a central role, it is important to ensure transgene functionality over the entire plant life during a field season. Indeed, the strong transgene silencing effects we saw in our lines can be the result of an orchestrated combination of different transcriptional and posttranscriptional effects, which together contribute to the downregulation of the transgene. Since gene expression levels might not be comparable among different (particularly, senescing) plant stages, the survey of the cytosine methylation levels was the more appropriate method to visualize changes during development. Comparable analysis of the timing of gene silencing in chicken cells indicated that histone hypoacetylation and transcriptional shutdown occurs even before the promoter shows hypermethylation [106].

### Inhibition of transgene silencing

Cytidine analogs and methytransferase blockers are commonly used treatments to prevent gene silencing in cell cultures. These chemicals can inhibit the transgene methylation process and have been successfully applied in plant, as well as in animal cell cultures [23,91,107]. However, a treatment of cell cultures differs substantially from that of an intact organism. The stable co-expression of silencing inhibitors in *N. benthamiana* and *N. tabacum* plants resulted in much higher transgene expression levels, but both plant species suffered from abnormal growth and altered leaf morphologies, which would invalidate their use in ecological experiments [68,108]. Although plants are surprisingly able to tolerate even mutations in genes of the DNA methylation pathway (e.g. *methyltransferase*1 mutants are embryolethal in mammals), the knockdown of the expression of these genes leads to the accumulation of developmental abnormalities [109,110]. The gene silencing machinery is an important part of the gene regulatory mechanism and their disturbance has global negative effects on development [111]. To date, there is no nuanced method available of selectively recovering only a single silenced transgene without causing substantial collateral damage to genome-wide methylation patterns.

### Reactivation of transgene expression through cell culture to rescue phenotypes for ecological research

The cell culture step of the plant transformation process is a common source of unintended side effects [70,112]. The somaclonal variations that result from the de- and re-differentiation steps of cell culturing can be of genetic or epigenetic origin. Since DNA methylation patterns were highly variable among regenerated plants, an altered DNA methylation machinery during cell culture conditions had been suggested [69,112,113]. Most studies in different plant species found a genome-wide trend towards hypomethylation after a tissue culture step with even the possibility of restoring the activity of a former deactivated transgene [101,114,115]. Recently, an epigenome analyses in rice (*Oryza sativa*) revealed the details of the genome-wide loss of DNA methylation after regeneration [4]. We demonstrated for transgenic *N. attenuata* plants, that a secondary callus regeneration step could be used to recover transgene expression in the offspring of the regenerated plants. In this way, the desired gene expression levels could be achieved, even from plants with progressively silenced constructs (Figure 8, Figure 9). However, the transgene was re-silenced within most of the regenerants after two generations, highlighting the temporary character of the recovery. Regardless, the onset of gene silencing was successfully deferred for one generation with plants that produce many seeds, which provides a long-term source of material for further experiments. Similar attempts in gentian plants failed and the gene suppression persisted, probably because already silenced leaf tissue was used for the secondary regeneration [116]. Therefore we used hypocotyl tissue of T_2_ seedlings, which were still resistant and indicated a relative low methylation rate (Figure 6). We hypothesize, that a cell culture induced transgene recovery mainly functions by interfering with the somatic *de novo* methylation process, rather than actively demethylating a transgene. The offspring of the regenerants were phenotypically normal, making this method suitable for ecological research.

## Conclusions

There is considerable interest in the creation of transformed plant lines with stable and heritable phenotypes, but the dynamics of epigenetic mechanisms during plant development can lead to gradual changes within a single generation and “transgene half-life” could compromise long-term experiments. Overall, the regulation of cytosine methylation in vegetative tissue seems be more dynamic than previously thought. Unlike in animals in which the germline is sequestered, plants develop germ cells directly from somatic cells relative late in their life cycle. Any vegetative acquired change of the genome could therefore be potentially submitted to the offspring, giving plants the potential to flexibly adapt to a rapid changing environment [117,118]. Apparently epigenetic processes can play a much greater role in driving plant evolution than previously thought [43,44,119].

## Methods

### Construction of transformation vectors

For heterologous expression of antimicrobial peptides in *N. attenuata* altogether 11 different vectors were constructed [61]. Plants transformed with the vectors pSOL9PNA, pSOL9ICE and pSOL9FAB were analyzed here in more detail. The different antimicrobial peptide coding genes were selected from the PhytAMP database (http://phytamp.pfba-lab-tun.org/) [120] and were synthesized in sequential PCR reactions with overlapping 40 bp primers. Full length synthesized genes were cloned in pSOL9 binary plant transformation vectors consisting of a *hygromycin phosphotransferase II* (*hpt*II) gene as a selectable marker under a *nopaline synthase* promoter (NOS) and the gene of interest (GOI) under a cauliflower mosaic virus promoter (35S) [61]. Transgenic “PNA” plants expressed an antifungal peptide (hevein) from the Japanese morning glory *Ipomoea nil* (synthetic gene similar to the Pn-AFP2 precursor [GenBank:U40076]). Transgenic “ICE” plants expressed an antimicrobial peptide (knottin) from the common ice plant *Mesembryanthemum crystallinum* (synthetic gene identical to the Mc-AMP1 precursor [GenBank:AF069321]) and transgenic “FAB” plants expressed an antimicrobial peptide (fabatin) from the broad bean *Vicia faba* (synthetic gene similar to the fabatin-1 precursor [GenBank:EU920043]). The sequences of the PNA and FAB constructs were manually adapted to the codon usage table of *N. tabacum* (http://gcua.schoedl.de/).

### Plant transformation and line screening

*N. attenuata* Torr. ex S. Watson seeds were originally collected in 1988 from a natural population at the DI Ranch in Southwestern Utah. Wild-type seeds from the 30th inbreed generation were used for the construction of transgenic plants and as WT controls in all experiments. Plant transformation was performed by *Agrobacterium tumefaciens*-mediated gene transfer as previously described [121]. Explant cultures were regenerated from elongated hypocotyl tissue and the selection for correct T-DNA integrations was performed on phytagel-based media supplemented with 20 mg/L hygromycin B (Duchefa). For germination seeds were sterilized for 5 min with a 2% (w/v) aqueous solution of sodium dichloroisocyanuric acid (DCCS) and treated for 1 h with 0.1 M gibberelic acid (GA_3_) in 50 × diluted liquid smoke solution (House of Herbs). At least 60 seedlings per plant were germinated on Gamborg’s B5 (GB5) Medium (Duchefa) supplemented with 35 mg/L hygromycin B (Duchefa) and incubated in a growth chamber (Percival, day 16 h 26°C, night 8 h 24°C). After 10 days the segregation rate (% of sensitive seedlings) was determined and resistant seedlings transferred to the glasshouse under constant temperature and light conditions (day 16 h 26-28°C, night 8 h 22-24°C). Since *N. attenuata* is self-compatible, the collected seeds result generally from self-pollination, except if crossings with different lines are indicated. For crossings, the flowers were antherectomized before opening and hand-pollinated using pollen from either homozygous transgenic or wild-type plants. Independent overexpression plant lines used in this study were: PNA 1 (A-09-678), PNA 3 (A-09-768), PNA 6 (A-09-792), PNA 8 (A-09-823), PNA 9 (A-09-825), PNA 10 (A-09-826), ICE 1 (A-09-653), ICE 4 (A-09-702), ICE 6 (A-09-748), ICE 10 (A-09-807), FAB 1 (A-09-662), FAB 5 (A-09-855), FAB 6 (A-09-857), FAB 9 (A-09-865), FAB 10 (A-09-866). The plant generations were indicated within the line number as follows: T_1_ seeds or plants have only the line number (e.g. PNA 8), T_2_ seeds were indicated by an extra number to identify the plant from which seeds were collected from (e.g. PNA 8.6), T_3_ seeds were additionally numbered (e.g. PNA 8.6.1 etc.). Two lines harboring an inverted repeat construct for silencing the expression of *N. attenuata acetyl-CoA-transferase* 1 (*acx*1), ir-ACX1 (A-07–466-1) and ir-ACX1 (A-07-468-3), were described in [37].

### Genomic DNA isolation

Genomic DNA was isolated with a modified hexadecyltrimethylammonium bromide (CTAB) method described in [122]. For Southern blotting 15 day old seedlings were ground in liquid nitrogen to a fine powder and 300 mg used for DNA isolation. The quality and concentration was estimated by agarose gel electrophoresis. For bisulfite sequencing gDNA was isolated from cotyledons and first true leaves of seedlings 15 days post germination (15 dpg), leaves of rosette-stage plants (30 dpg), cauline leaves of elongating plants (45 dpg) and cauline leaves of flowering plants (60 dpg) (see Figure 6A for illustration). The last three time points were successively sampled from the same plants. Materials from 5 biological replicates were pooled, ground in liquid nitrogen to a fine powder and 300 mg used for DNA isolation. For the isolation of DNA from elongated plants (45 and 60 dpg) a modified buffer with higher salt concentration was used (2% CTAB, 100 mM Tris–HCl [pH 8.0], 20 mM EDTA [pH 8.0], 2.2 M NaCl, 2% PVPP [Mr 40.000], 10 mM ascorbic acid). The amount and quality of DNA was estimated on a Nanodrop spectrophotometer (Thermo Scientific).

### Southern blot analysis

A total amount of 6 μg gDNA was digested overnight at 37°C with 140 U *Eco*RV and *Xba*I (New England Biolabs) in independent reactions, each enzyme providing only one restriction site within the T-DNA of the binary vector (indicated in Figure 5A). The digested DNA was separated on a 1% (w/v) agarose gel for 17 h at 23 Volt. DNA was blotted overnight onto a Gene Screen Plus Hybridization Transfer Membrane (Perkin-Elmer) using the capillary transfer method. A gene specific probe for the *hpt*II gene was amplified with the primer pair HYG1-18 (5′-CCGGATCGGACGATTGCG-3′) and HYG2-18 (5′-CTGACGGACAATGGCCGC-3′) [61] and radiolabeled with [α-^32^P] dCTP (Perkin-Elmer) using the Rediprime II DNA Labeling System (GE Healthcare) according to the manufacturer′s instructions.

### RNA isolation and qRT-PCR

Tissue was harvested from rosette-stage leaves and ground in liquid nitrogen to a fine powder. RNA isolation was performed with a salt precipitation method modified from the US patent of Gentra Systems, Inc. publication No. 5973137 [123] and adapted for *N. attenuata* tissue. Approximately 150–300 mg ground and frozen tissue was dissolved in 900 μL cell lysis buffer (2% [w/v] sodium dodecyl sulfate, 77 mM [tri-] sodium citrate, 132 mM citric acid, 10 mM ethylenediaminetetraacetic acid) and shortly mixed. Per sample 300 μL protein precipitation buffer (4 M NaCl, 19 mM [tri-] sodium citrate, 33 mM citric acid) was added and the tubes inverted ten times. Samples were incubated on ice for 5 min and centrifuged at room temperature in a table top centrifuge (5 min at 16.100 g). The supernatant was collected and extracted with 500 μL chloroform:isoamylalcohol mix (24:1 v/v). After centrifugation (3 min at 16.100 g) the upper aqueous phase was collected and nucleic acids precipitated with 1 volume isopropanol for 15 min at room temperature. Nucleic acids were pelleted in a table top centrifuge (3 min at 16.100 g), washed twice with 400 μL 70% (v/v) ethanol and air dried for 5 min. The final pellet was dissolved in 50 μL nuclease free water (Ambion / Life technologies). The nucleic acid was DNAse-treated using the TURBO DNA-free kit (Ambion / Life Technologies) according to the manufacturer’s instructions. Quality and amount of the remaining RNA was determined using a 1% (w/v) agarose gel and a Nanodrop spectrophotometer (Thermo Scientific). The absence of genomic DNA was tested with 20 ng RNA in a 35 cycle PCR programm (94°C for 1 min, 35 cycles of 94°C for 30 s, 60°C for 30 s, 72°C for 30 s) with the same primers as for qRT-PCR. 4 μg of total RNA was reverse transcribed with oligo(dT)_18_ primers (Fermentas) and the SuperScript II reverse transcriptase enzyme (Invitrogen / Life Technologies). Quantitative Real Time-PCR (qRT-PCR) was performed with 1:10 diluted cDNA (20 ng) on a Mx3005P QPCR System (Stratagene) with either a SYBR Green based PCR Master Mix (Applied Biosystems / Life Technologies) or a qPCR Core kit for SYBR Green (Eurogentec). For amplification the following primers were used: ICE-94F (5′-AATGGAAAAGGATGTCGAGAGG-3′), ICE-167R (5′-CATCCAACCTGACGGTAACAGAA-3′), PNA-86F (5′-GGAGACAAGCTAGTGGGAGGC-3′), PNA-154R (5′-TGGAGCCACAGTAGCCCC-3′), FAB-111F (5′-CAGGTTTAATGGACCATGCTTG-3′), FAB-184R (5′-CACCACCTTTGTAACCTTCTCCC-3′). The used program was 95°C for 10 min followed by 40 cycles of 95°C for 15 s, 60°C for 1 min and 1 cycle of 95°C for 15 s, 60°C for 30 s, 95°C for 15 s as dissociation curve. For relative gene expression analysis the comparative Δ cycle threshold method (Δ*C*_T_ ) was used. Gene expression was shown as log_2_ (Δ*C*_T_) relative to *N. attenuata* actin as the reference gene (Actin-F1 5′-GGTCGTACCACCGGTATTGTG-3′ and Actin-R1 5′-GTCAAGACGGAGAATGGCATG-3′) [61].

### Bisulfite genomic sequencing

DNA methylation analysis was performed by the bisulfite sequencing method [124]. The bisulfite conversion was performed using the EpiTect Bisulfite kit (Qiagen) according to the manufacturer’s instructions. A total of 1 μg gDNA was converted for 5 h with the following program 95°C for 5 min, 60°C for 25 min, 95°C for 5 min, 60°C for 85 min, 95°C for 5 min, 60°C for 175 min. The target sequences were amplified from the converted DNA with 0,05 U/μL JumpStart Taq DNA Polymerase with the provided reaction buffer (Sigma-Aldrich), 200 μM dNTP Mix (Fermentas) and 0,5 μM of the following primer: MetCNOSR5 (5′-AGATYYGGTGYAGATTATTTGGATTGA-3′) and MetCNOSF6 (5′-TTARRTCCTCTATTTRAATCTTTRACTCC-3′) for a 294 bp fragment of the NOS promoter (−40 to −333 bp before the start codon) and MetC35SF2 (5′-AGGGYAATTGAGAYTTTTTAATAAAGGG-3′) and MetC35SRPNA2 (5′-CAARARAACAATAAACATAATACARTATTTCATCTC-3′) or MetC35SRICE2 (5′-ATTTCARCAAAAAARATRAAACCTTAACCATCTC-3′) for a 346 bp fragment of the CaMV 35S promoter (−1 to −346 bp before the start codon). Primers were designed using Methprimer software (http://www.urogene.org/methprimer/) and Kismeth [110] and manually adapted according to [125] and [126], to avoid amplification-bias of non-converted DNA. Cycle parameters used were 94°C for 1 min followed by 35 cycles with 94°C for 30 s, 53°C for 30 s, 72°C for 30 s and a final step with 72°C for 5 min. PCR products were gel excised and purified with the NucleoSpin Extract II kit (Macherey-Nagel) and cloned into pGEM-T Easy vector system (Promega). Plasmids of individual picked clones were isolated with NucleoSpin Plasmid Kit (Macherey-Nagel). Sequencing was performed with the BigDye Terminator mix v3.1 (Applied Biosystems) supplemented with 5% dimethyl sulfoxide (DMSO). Sequences were manually trimmed and the data analysis performed with the online tools CyMATE (http://cymate.org/) [127] and MethTools 2.0 (http://methdb.igh.cnrs.fr/methtools/) [128]. Nucleotide frequencies at CHH positions were graphical illustrated with WebLogo 3 (http://weblogo.threeplusone.com/) [129]. For the 35S promoter methylation kinetic a minimum of 10–12 individual clones per sample were analyzed.

### Secondary callus regeneration

Homozygous seedlings of the lines PNA 1.2, ICE 4.4 and ir-ACX1 (A-07-466-1 and A-07-468-3) were chosen for secondary callus regeneration. T_2_ stage seedlings (still resistant) were grown for 10 days on GB5 media supplemented with hygromycin B (35 mg/L). The hypocotyls were cut in small pieces as done for the normal plant transformation procedure but without dipping the scalpel in *Agrobacterium* suspension. The explant cultures were grown into a callus and regenerated as previously described [121]. Fully regenerated plants were grown in pots in the glasshouse for self-pollination and seed production. Secondary regenerated lines originating from PNA 1.2 seedlings were A-11-xxx (188, 189, 190, 191, 193, 194, 272, 274, 275, 276, 277, 278, 286, 288, 308, 327, 329 and 330). Secondary regenerated plants originating from ICE 4.4 seedlings were A-11-xxx (195, 196, 199, 200, 201, 202, 268, 269, 270, 271, 307 and 328). Secondary regenerated lines originating from ir-ACX1 (A-07-466-1) seedlings were: A-11-xxx (170, 171, 172, 173, 174, 175, 176, 177, 178, 263, 264, 265, 281 and 283) and from ir-ACX1 (A-07-468-3) A-11-xxx (179, 180, 181, 183, 184, 185, 266 and 282). The first seed generation (T_3_) from the regenerants were germinated on hygromycin containing media and seedlings with 0% sensitivity were brought to the glasshouse for RNA isolation and further propagation to test the subsequent generation (T_4_) for resistance.

### Jasmonic acid extraction and analysis

Leaves at nodes +1 [130] from rosette-stage (30 days old) plants were wounded by rolling a fabric pattern wheel three times on each side of the midvein and the wounds were supplied immediately with 20 μL of 1:5 (v/v) diluted oral secretion of *Manduca sexta*. Leaf tissue was collected 60 min after the treatment and was frozen immediately in liquid nitrogen for subsequent analysis. Jasmonic acid was extracted and analyzed as described in [35].

## Competing interests

The authors declare that they have no competing interests.

## Authors’ contributions

AW planned and performed the experiments, analyzed the data and wrote the manuscript. MK performed all experiments with ir-ACX1 lines. ITB participated in the design of the study and revised the manuscript. All authors read and approved the final manuscript.

## Supplementary Material

Additional file 1 Fold difference of transgene expression in consecutive generations. Click here for file

Additional file 2 **NOS promoter methylation between sensitive and resistant seedlings of line ICE 10.1.A**, Methylation status of the NOS promoter among isogenic seedlings from line ICE 10.1. Different methylation sites (CG, CHG and CHH) were indicated by different colors. Analysis was performed by CyMATE [127]. **B**. Phenotypes of 10-day-old seedlings used for DNA isolation and bisulfite conversion. Isogenic seedlings of line ICE 10.1.2 were divided into sensitive and resistant seedlings and analyzed separately. Mean methylation rate from five clones is shown for the individual methylation sites (CG, CHG and CHH). (± SEM, n = 5 clones). Click here for file

Additional file 3 **Detail of 35S promoter methylation analysis of individual clones.** Tissue harvested 15, 30, 45 and 60 days post germination of lines ICE 4.4 (T_2_) and ICE 4.4.1 (T_3_); ICE 1.1 (T_2_), ICE 1.1.1 (T_3_) and ICE 1.1.1.1 (T_4_); PNA 1.2 (T_2_) and PNA 1.2.1 (T_3_); PNA 10.1 (T_2_) and PNA 10.1.1 (T_3_); PNA 8.6 (T_2_) and PNA 8.6.1 (T_3_). Click here for file

Additional file 4 **Sequence preference in CHH methylation sites.** The nucleotide composition of 8-mer sequences around the CHH sites (methylated cytosine in the fifths position) divided in groups with low methylation (0–9%), medium methylation (10–49%) and high methylation (50–100%) frequencies. The pooled frequency data of lines ICE 4.4.1, PNA 1.2.1 and PNA 10.1.1 derived from one time point (60 dpg T_3_). The logo graphically illustrates the sequence enrichment at particular positions around the methylation site. Maximum sequence conservation is 2 bit, no nucleotide preference is 0 bit. Figures were made with WebLogo 3 [129]. Click here for file

Additional file 5 **Inheritance of the silenced allele after reciprocal crossing with wild-type.** The hybrid offspring (hemizygous to the transgene) should be theoretically fully resistant to hygromycin B. The silenced state of the transgene was equally distributed to subsequent generations. **A**, Percentage of sensitive seedlings after crossing (± SD, n = 3 plants). **B**, Phenotypes of seedlings on hygromycin B containing GB5 media. Click here for file

Additional file 6 **Transgene silencing in line ir-ACX1.A**, Jasmonic acid accumulation 1 h after wound and oral secretion treatment in rosette leaves of ir-ACX1 and wild-type plants. The T_3_ generation of ir-ACX1 lost their capacity to suppress jasmonic acid accumulation [37]. **B**, The T_3_ seedlings from line ir-ACX1 (A-07-468) developed sensitivity to hygromycin B. **C**, Transgene activity indicated by jasmonic acid accumulation determined in wound and oral secretion treated leaves of secondary regenerated ir-ACX1 lines. A reduced accumulation of jasmonic acid after wounding compared to wild-type (WT) indicated a functional IR-construct. **D**, Hygromycin sensitivity of T_4_ seedlings (direct descendants of the plants used for wound treatment) indicates an ongoing silencing process (±SD, n = 3 plants). Click here for file

Additional file 7 **Phenotypes after secondary regeneration. A**, Photographs of T_3_ seedlings collected from secondary regenerants of line ICE 4.4 and PNA 1.2. Cell culture-induced variations resulted in variegated pattern of sensitivity on hygromycin B containing GB5 media. **B**, Photographs of T_4_ generation seedlings collected from fully resistant secondary regenerated plants. As positive and negative controls conventional propagated T_4_ seedlings are shown. Click here for file
