# Supplementary material for: Progressive 35S promoter methylation increases rapidly during vegetative development in transgenic Nicotiana attenuata plants
Source: BMC Plant Biol. 2013 Jul 9;13:99. doi: 10.1186/1471-2229-13-99 (PMC3716894; doi:10.1186/1471-2229-13-99)
Supplement: Additional file 1 — Fold difference of transgene expression in consecutive generations. [file 1471-2229-13-99-S1.docx]

## Additional file 1: Fold difference of transgene expression in consecutive generations

|  |  | ΔC_T_ (C_T_ actin – C_T_ *goi*) | ΔΔC_T_ (T_3_– T_2/control_) | Fold difference (2^–ΔΔCt^) |
| --- | --- | --- | --- | --- |
| T_2_ | PNA 1.2 | 2.91 ± 1.60 | 0.00 ± 1.60 | 1.7 (0.3 – 3.0) |
| T_3_ | PNA 1.2.1 | -4.52 ± 1.45 | -7.43 ± 1.45 | 267.6 (62.9 – 472.2) |
|  |  |  |  |  |
| T_2_ | PNA 10.1 | 2.51 ± 1.23 | 0.00 ± 1.23 | 1.4 (0.4 – 2.3) |
| T_3_ | PNA 10.1.1 | -4.59 ± 1.44 | -7.10 ± 1.44 | 210.4 (50.6 – 370.1) |
|  |  |  |  |  |
| T_3_ | PNA 8.6.1 | 4.56 ± 0.34 | 0.00 ± 0.34 | 1.0 (0.8 – 1.3) |
| T_3_ | PNA 1.2.1 |  | -9.07 ± 1.45 | 836.4 (196.6 – 1476.3) |
| T_3_ | PNA 10.1.1 |  | -9.15 ± 1.44 | 871.8 (209.8 – 1533.8) |
|  |  |  |  |  |
| T_2_ | ICE4.4 | 0.37 ± 2.82 | 0.00 ± 2.82 | 3.6 (0.1 – 7.1) |
| T_3_ | ICE4.4.1 | -4.36 ± 1.47 | -4.73 ± 1.47 | 41.4 (9.6 – 73.2) |
|  |  |  |  |  |
| T_3_ | ICE 1.1.1 | 3.74 ± 0.40 | 0.00 ± 0.40 | 1.0 (0.8 – 1.3) |
| T_3_ | ICE4.4.1 |  | -8.10 ± 1.47 | 427.7 (98.8 – 756.6) |
