# Supplementary material for: Progressive 35S promoter methylation increases rapidly during vegetative development in transgenic Nicotiana attenuata plants
Source: BMC Plant Biol. 2013 Jul 9;13:99. doi: 10.1186/1471-2229-13-99 (PMC3716894; doi:10.1186/1471-2229-13-99)
Supplement: Additional file 2 — NOS promoter methylation between sensitive and resistant seedlings of line ICE 10.1.A, Methylation status of the NOS promoter among isogenic seedlings from line ICE 10.1. Different methylation sites (CG, CHG and CHH) were indicated by different colors. Analysis was performed by CyMATE [127]. B. Phenotypes of 10-day-old seedlings used for DNA isolation and bisulfite conversion. Isogenic seedlings of line ICE 10.1.2 were divided into sensitive and resistant seedlings and analyzed separately. Mean methylation rate from five clones is shown for the individual methylation sites (CG, CHG and CHH). (± SEM, n = 5 clones). [file 1471-2229-13-99-S2.pdf]

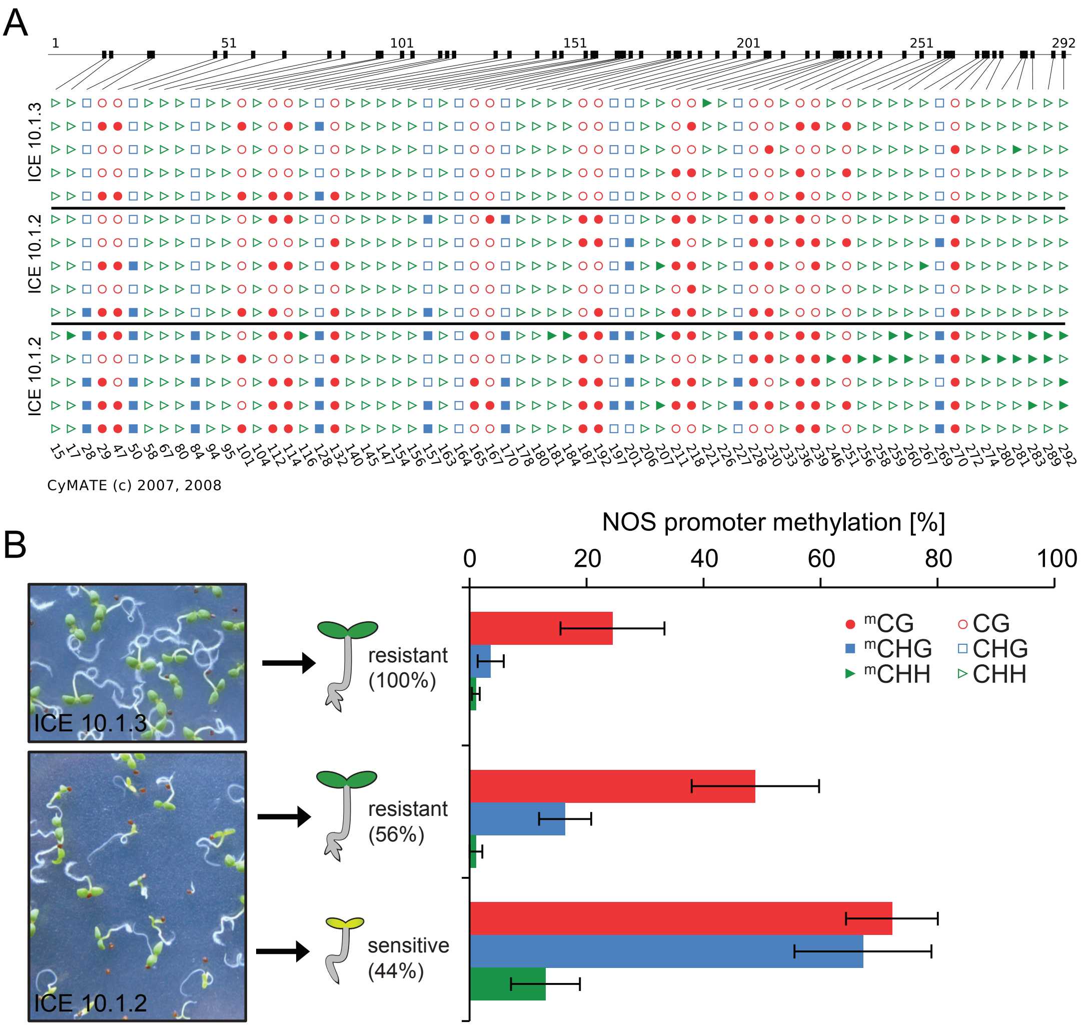

Additional file 2: NOS promoter methylation between sensitive and resistant seedlings of line ICE 10.1
