# Supplementary material for: Progressive 35S promoter methylation increases rapidly during vegetative development in transgenic Nicotiana attenuata plants
Source: BMC Plant Biol. 2013 Jul 9;13:99. doi: 10.1186/1471-2229-13-99 (PMC3716894; doi:10.1186/1471-2229-13-99)
Supplement: Additional file 3 — Detail of 35S promoter methylation analysis of individual clones. Tissue harvested 15, 30, 45 and 60 days post germination of lines ICE 4.4 (T2) and ICE 4.4.1 (T3); ICE 1.1 (T2), ICE 1.1.1 (T3) and ICE 1.1.1.1 (T4); PNA 1.2 (T2) and PNA 1.2.1 (T3); PNA 10.1 (T2) and PNA 10.1.1 (T3); PNA 8.6 (T2) and PNA 8.6.1 (T3). [file 1471-2229-13-99-S3.pdf]

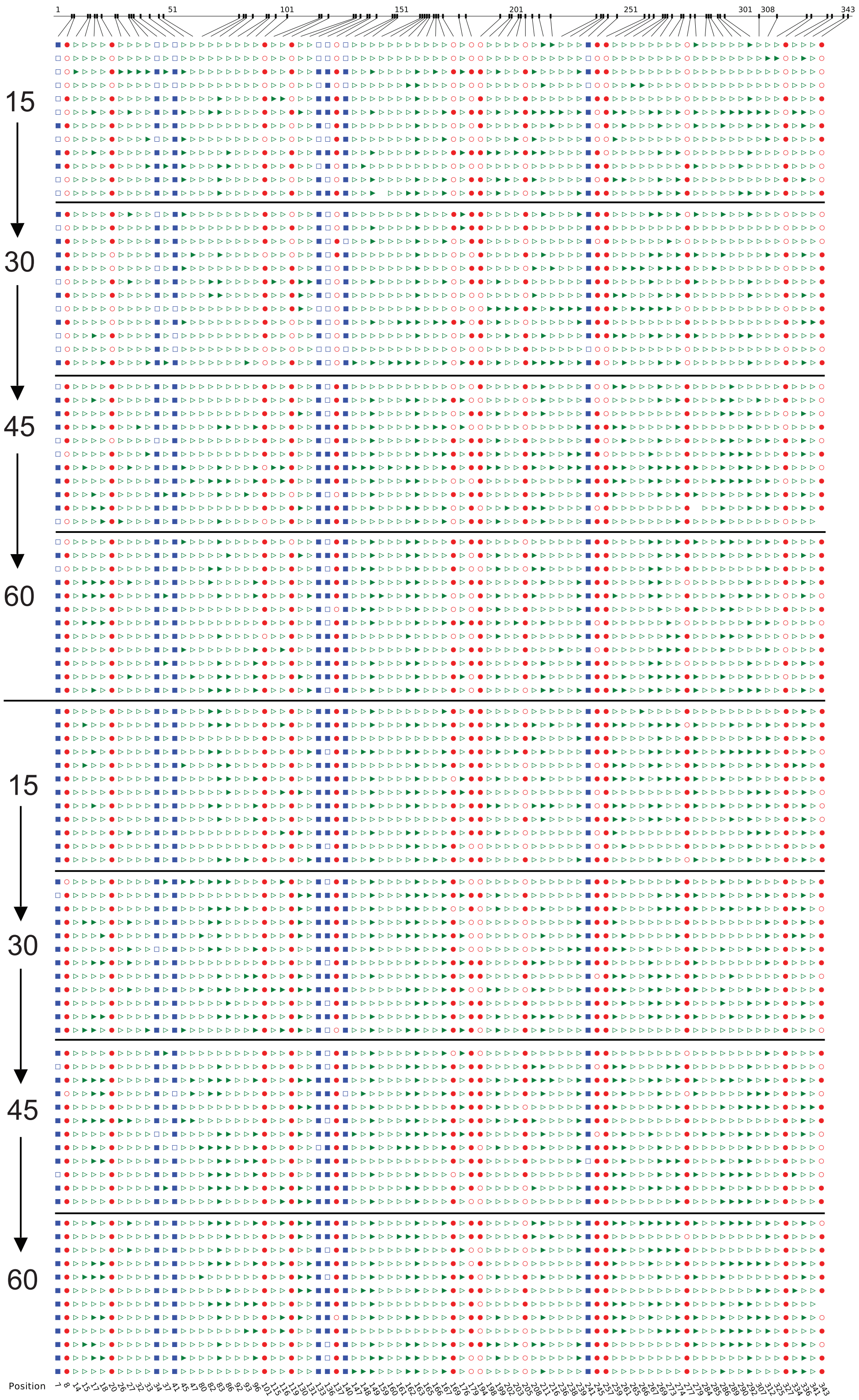

Additional file 3: 35S promoter methylation analysis of individual clones from tissue harvested 15, 30, 45 and 60 days post germination of line ICE 4.4 (T_2) and ICE 4.4.1 (T_3).

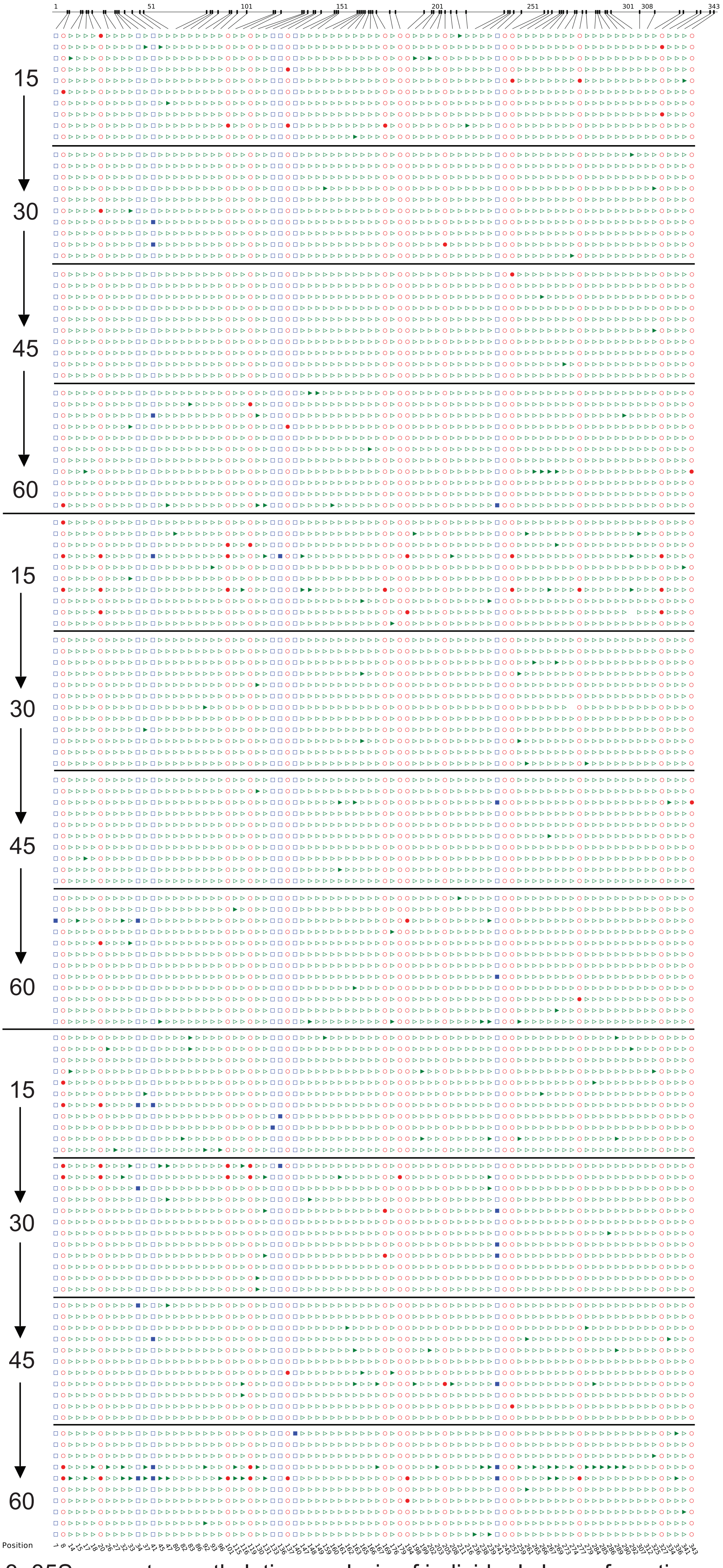

Additional file 3: 35S promoter methylation analysis of individual clones from tissue harvested 15, 30, 45 and 60 days post germination of line ICE 1.1 (T_2), ICE 1.1.1 (T_3) and ICE 1.1.1.1 (T_4).

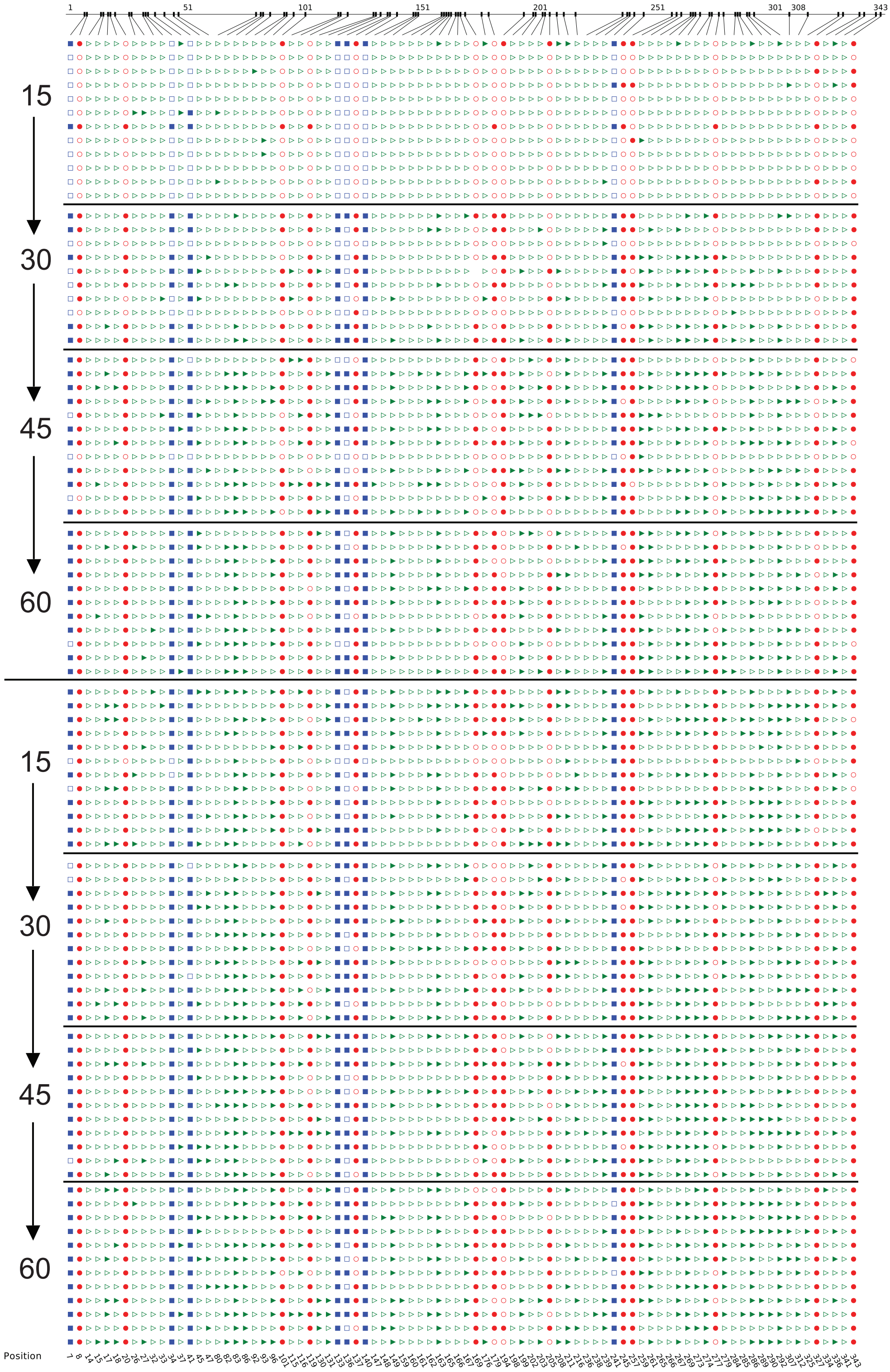

Additional file 3: 35S promoter methylation analysis of individual clones from tissue harvested 15, 30, 45 and 60 days post germination of line PNA 1.2 (T_2) and PNA 1.2.1 (T_3).

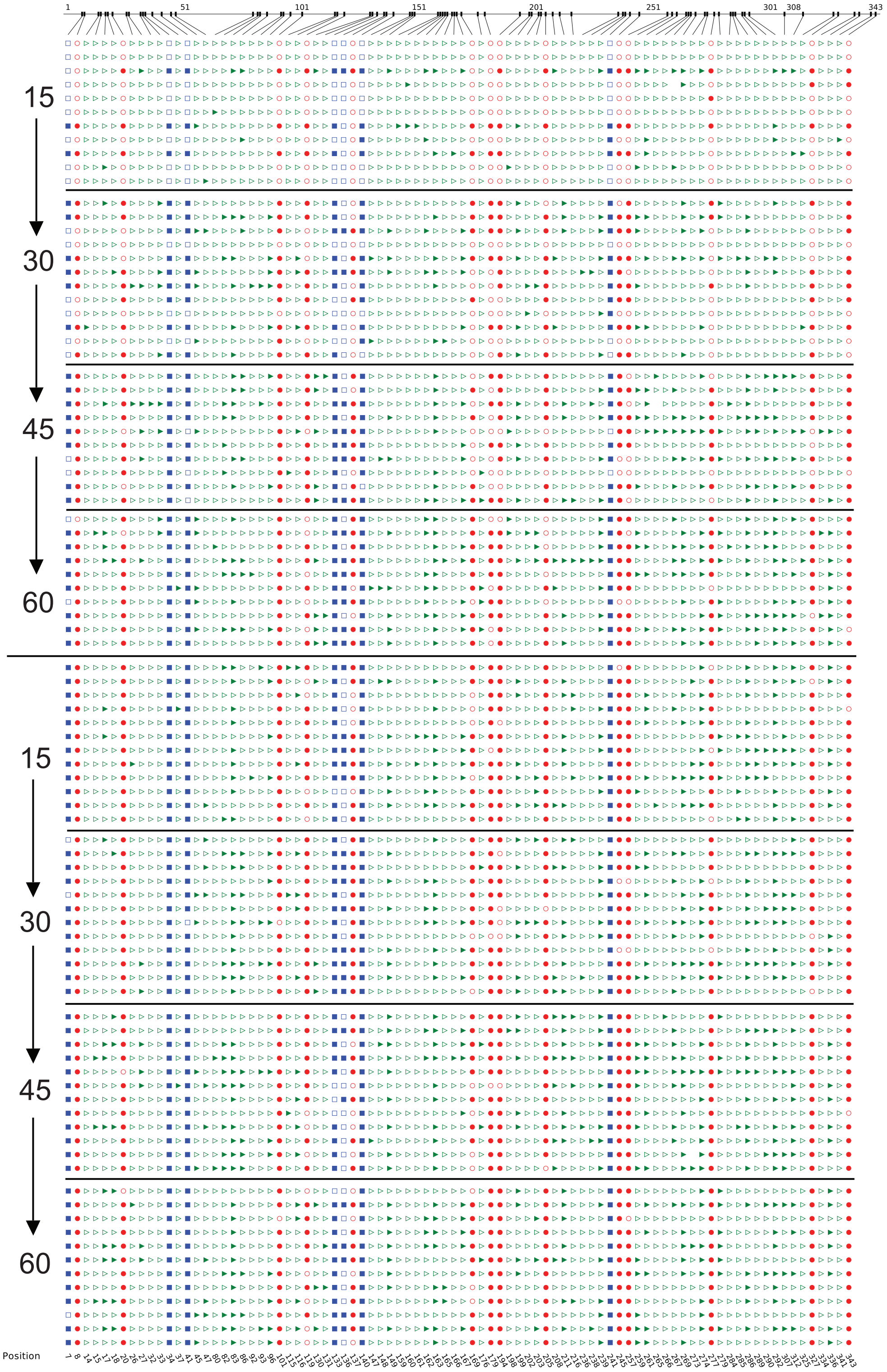

Additional file 3: 35S promoter methylation analysis of individual clones from tissue harvested 15, 30, 45 and 60 days post germination of line PNA 10.1 (T_2) and PNA 10.1.1 (T_3).

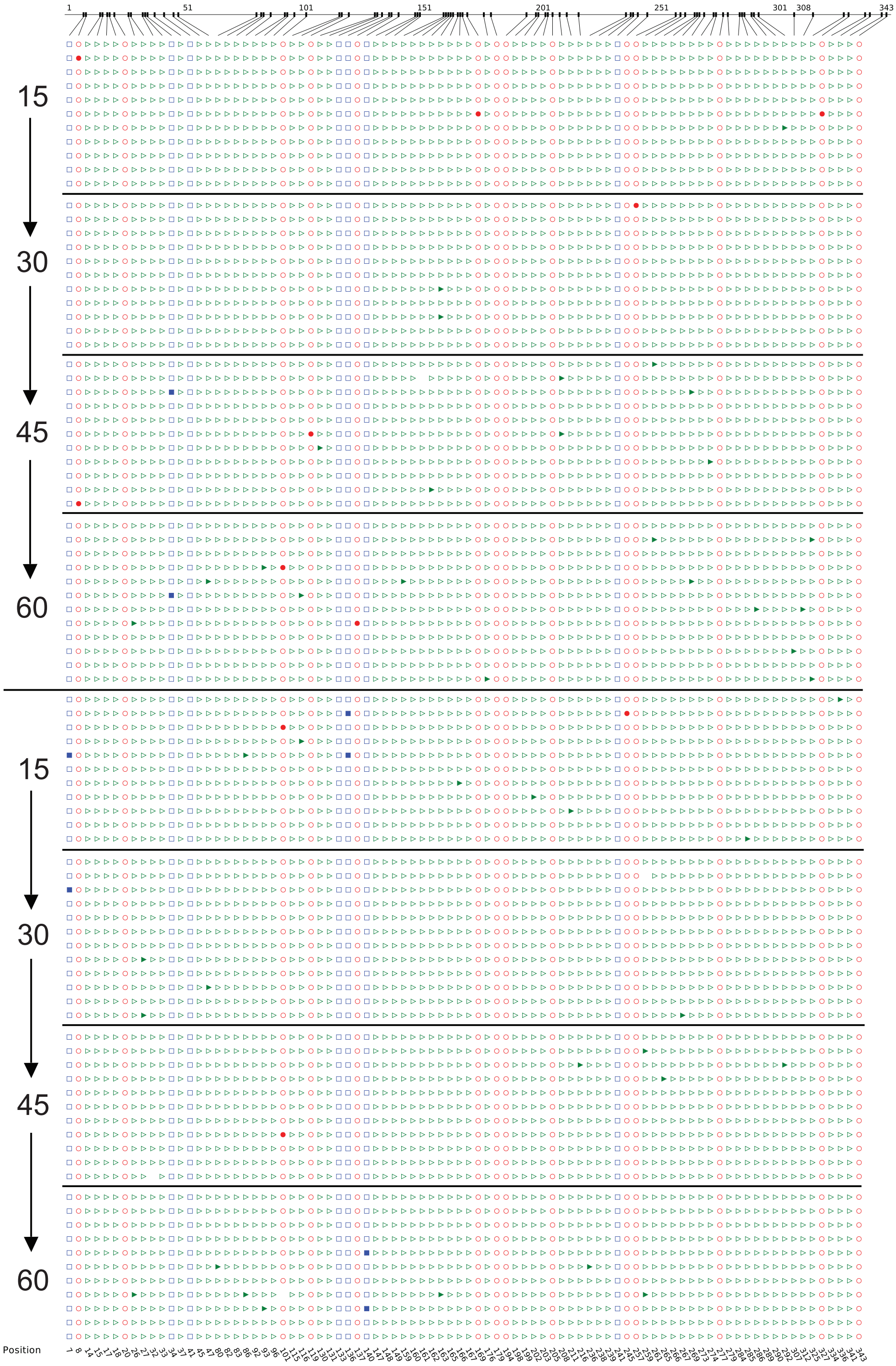

Additional file 3: 35S promoter methylation analysis of individual clones from tissue harvested 15, 30, 45 and 60 days post germination of line PNA 8.6 (T_2) and PNA 8.6.1 (T_3).
