# Supplementary figures and images for: Progressive 35S promoter methylation increases rapidly during vegetative development in transgenic Nicotiana attenuata plants
Source: BMC Plant Biol. 2013 Jul 9;13:99. doi: 10.1186/1471-2229-13-99 (PMC3716894; doi:10.1186/1471-2229-13-99)

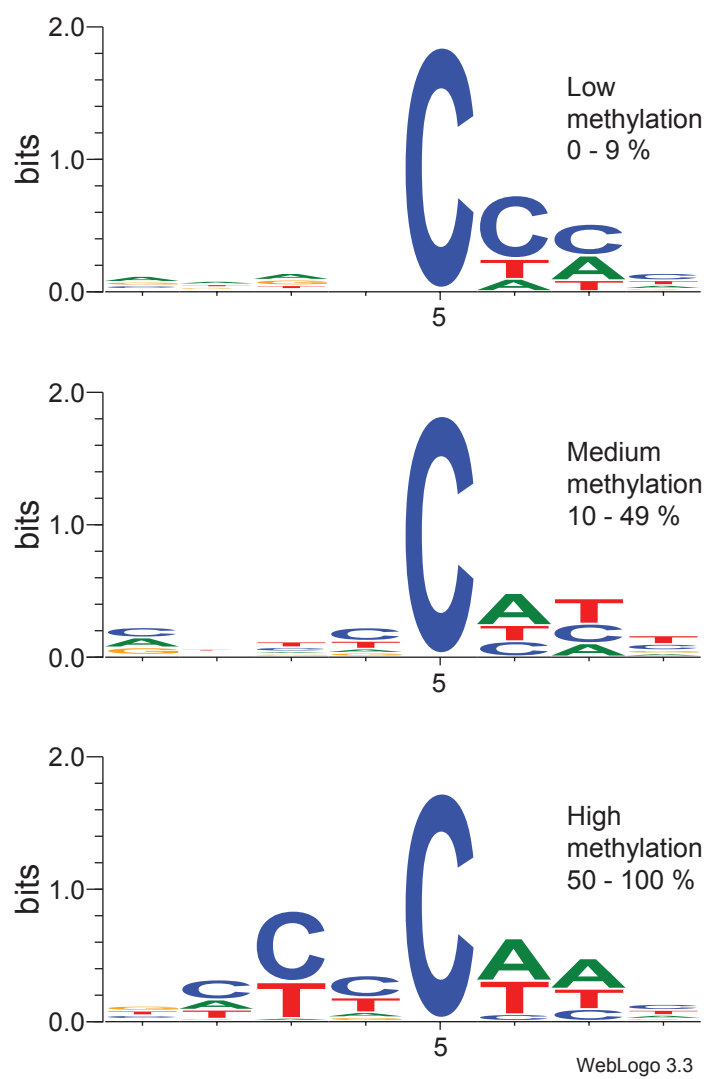

Additional file 4: Sequence preference in CHH methylation sites

Supplement: Additional file 4 — Sequence preference in CHH methylation sites. The nucleotide composition of 8-mer sequences around the CHH sites (methylated cytosine in the fifths position) divided in groups with low methylation (0–9%), medium methylation (10–49%) and high methylation (50–100%) frequencies. The pooled frequency data of lines ICE 4.4.1, PNA 1.2.1 and PNA 10.1.1 derived from one time point (60 dpg T3). The logo graphically illustrates the sequence enrichment at particular positions around the methylation site. Maximum sequence conservation is 2 bit, no nucleotide preference is 0 bit. Figures were made with WebLogo 3 [129]. [file 1471-2229-13-99-S4.pdf]

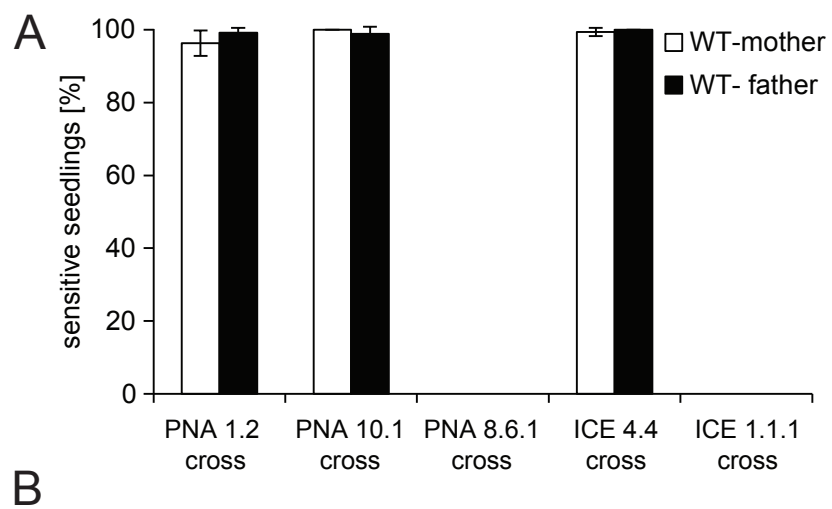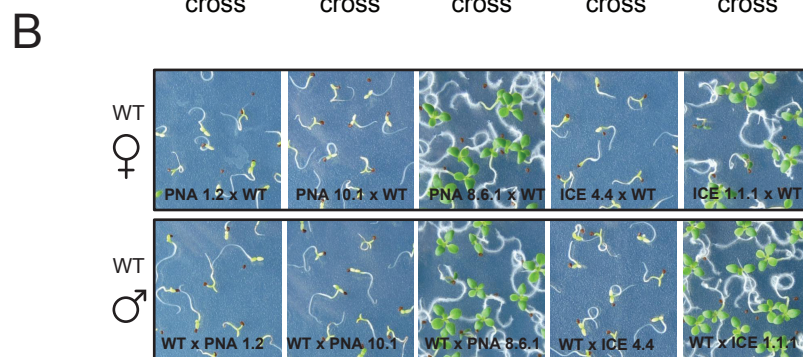

Additional file 5: Inheritance of the silenced allele after reciprocal crossing with wild-type

Supplement: Additional file 5 — Inheritance of the silenced allele after reciprocal crossing with wild-type. The hybrid offspring (hemizygous to the transgene) should be theoretically fully resistant to hygromycin B. The silenced state of the transgene was equally distributed to subsequent generations. A, Percentage of sensitive seedlings after crossing (± SD, n = 3 plants). B, Phenotypes of seedlings on hygromycin B containing GB5 media. [file 1471-2229-13-99-S5.pdf]

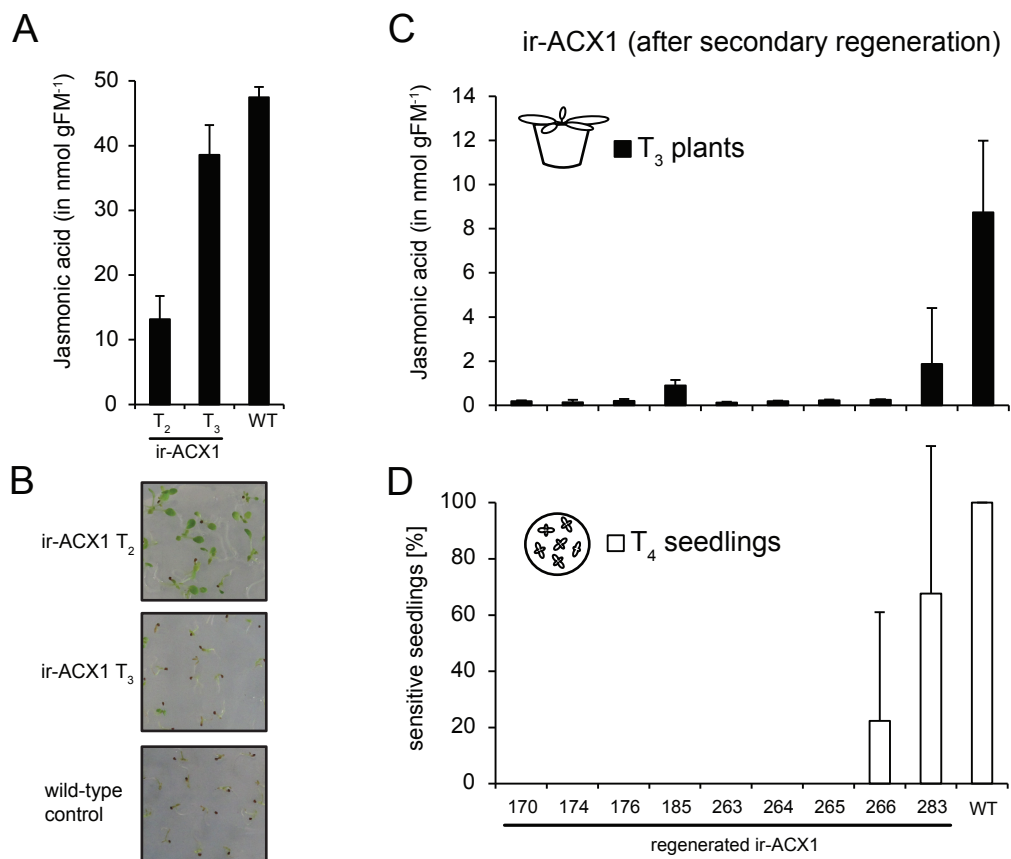

Additional file 6: Transgene silencing in line ir-ACX1

Supplement: Additional file 6 — Transgene silencing in line ir-ACX1.A, Jasmonic acid accumulation 1 h after wound and oral secretion treatment in rosette leaves of ir-ACX1 and wild-type plants. The T3 generation of ir-ACX1 lost their capacity to suppress jasmonic acid accumulation [37]. B, The T3 seedlings from line ir-ACX1 (A-07-468) developed sensitivity to hygromycin B. C, Transgene activity indicated by jasmonic acid accumulation determined in wound and oral secretion treated leaves of secondary regenerated ir-ACX1 lines. A reduced accumulation of jasmonic acid after wounding compared to wild-type (WT) indicated a functional IR-construct. D, Hygromycin sensitivity of T4 seedlings (direct descendants of the plants used for wound treatment) indicates an ongoing silencing process (±SD, n = 3 plants). [file 1471-2229-13-99-S6.pdf]
