# Supplementary material for: Progressive 35S promoter methylation increases rapidly during vegetative development in transgenic Nicotiana attenuata plants
Source: BMC Plant Biol. 2013 Jul 9;13:99. doi: 10.1186/1471-2229-13-99 (PMC3716894; doi:10.1186/1471-2229-13-99)
Supplement: Additional file 7 — Phenotypes after secondary regeneration. A, Photographs of T3 seedlings collected from secondary regenerants of line ICE 4.4 and PNA 1.2. Cell culture-induced variations resulted in variegated pattern of sensitivity on hygromycin B containing GB5 media. B, Photographs of T4 generation seedlings collected from fully resistant secondary regenerated plants. As positive and negative controls conventional propagated T4 seedlings are shown. [file 1471-2229-13-99-S7.pdf]

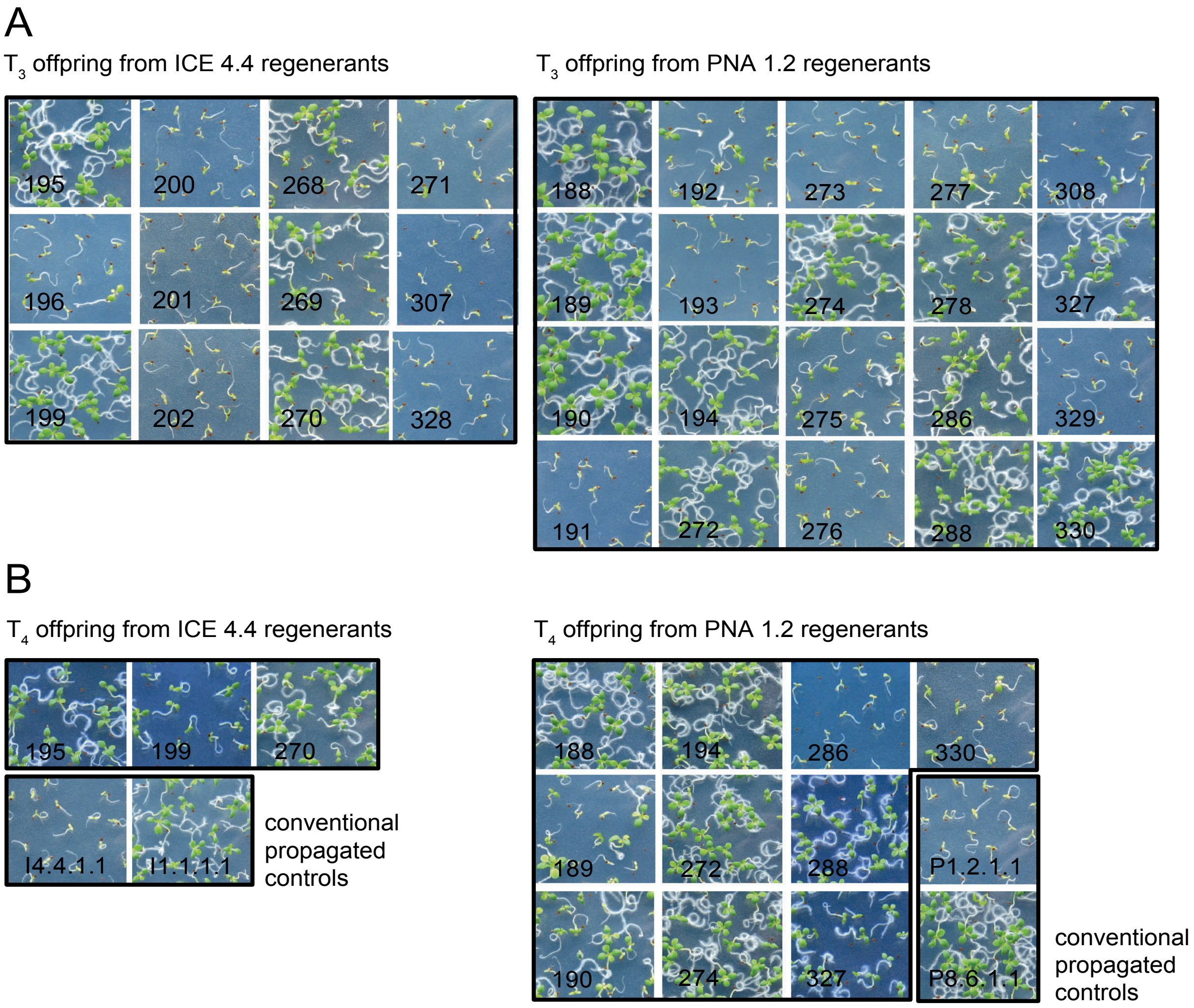

Additional file 7: Phenotypes after secondary regeneration
